# The Effect of Anthocyanins on Cognition: A Systematic Review and Meta-analysis of Randomized Clinical Trial Studies in Cognitively Impaired and Healthy Adults

**DOI:** 10.1007/s13668-024-00595-z

**Published:** 2025-01-29

**Authors:** Elnaz Lorzadeh, Katrina Weston-Green, Steven Roodenrys, Vinicius do Rosario, Katherine Kent, Karen Charlton

**Affiliations:** 1 School of Medical, Indigenous and Health Sciences, Faculty of Science, Medicine and Health, University of Wollongong NSW, Wollongong, 2522 Australia; 2 Molecular Horizons, University of Wollongong, Wollongong, NSW 2522 Australia; 3 School of Psychology, Faculty of Arts, Social Sciences and Humanities, University of Wollongong, Wollongong, NSW 2522 Australia

**Keywords:** Anthocyanin, Anthocyanin supplementation, Cognition, Cognitive domains, Meta analysis, Systematic review

## Abstract

**Purpose of the Review:**

Clinical trials suggest that dietary anthocyanins may enhance cognitive function. This systematic literature review and meta-analysis aimed to identify the effect of anthocyanin on cognition and mood in adults.

**Recent Findings:**

Using a random-effects model, Hedge’s g scores were calculated to estimate the effect size. Across 30 randomized controlled trials, fourteen (*n* = 733 participants) met the criteria for meta-analysis following PRISMA guidelines (Registration number: CRD42021279470). Qualitative synthesis showed improvements in multiple domains after anthocyanin intake: short-term memory, verbal learning and working memory, executive function, visual-spatial function, psychomotor skills, attention and semantic memory. Four of 15 studies reported significant mood improvements, including anti-fatigue and reduced anxiety and depression scores. However, there were no significant effects for working memory (Hedges’s g = -0.183, 95% CI = -0.407 to 0.041, *P* = 0.110), verbal learning (Hedges’s g = 0.054, 95% CI = -0.215 to 0.324, *P* = 0.69), immediate memory (Hedges’s g = 0.196, 95% CI = -0.242 to 0.633, *P* = 0.38) and delayed memory (Hedges’s g = -0.188, 95% CI = -0.629 to -0.252, *P* = 0.402) according to the meta-analysis.

**Summary:**

This review suggests potential benefits of anthocyanin intake on cognition and mood. However, in meta-analysis of 14 eligible studies, effects on working, immediate, delayed memory and verbal learning were not significant, likely due to study heterogeneity. Recommendations for future study designs are discussed.

**Supplementary Information:**

The online version contains supplementary material available at 10.1007/s13668-024-00595-z.

## Introduction

Cognitive function includes multiple domains such as attention, executive function, speed of processing, and memory [1]. Performance in these domains tends to increase from early childhood until adulthood, followed by a gradual decline after midlife (approximately 50yrs of age), with a sharper decline after the age of 70yrs [2, 3]. With an aging population, the prevalence of chronic diseases, such as diabetes, cancer, cardiovascular diseases, and neurodegenerative diseases including dementia, is rising [4, 5]. Hence, the early detection of cognitive impairment and identification of beneficial lifestyle behaviours, including a healthy diet, in the prevention and reduction of symptom severity is important [6, 7]. High consumption of fruits and vegetables as the primary source of antioxidants (vitamin C, B complex, and E, etc.), carotenoids, and polyphenols, has been associated with reduced risk of chronic disease and better cognitive performance in older adults [8–11]. Furthermore, evidence suggests that certain flavonoids, such as the subclass of anthocyanin, may delay the onset of Alzheimer’s disease and other cognitive deficits across multiple animal models [12, 13]. Aside from their antioxidant capacities, flavonoids appear to have neuroprotective and anti-inflammatory benefits in the brain [14, 15]. Emerging evidence from cohort studies suggests that moderate intakes of subclasses of flavonoids can be associated with lower incidence of dementia [16]. Specifically, anthocyanins, that are water-soluble plant pigments responsible for the deep red, blue and purple colour of fruits such as berries, cherries and plums, as well as some vegetables (e.g. red onion and red radish) and legumes (e.g. black beans) [17, 18], have shown to be particularly promising in terms of protecting and improving brain health [19–21].

The neuroprotective effects of anthocyanins are supported by evidence from *in-vitro* studies using fruit extracts, both as isolated anthocyanins or combinations of different anthocyanins, tested in neuronal primary cultures and cell lines. [22, 23]. Pre-clinical rodent studies support the *in-vitro* findings by showing promising effects of anthocyanins on various aspects of cognitive function, such as long-term memory [24], spatial-working memory [25] and object-recognition memory [26]. Recent systematic reviews of human studies that have assessed the effect of habitual blueberry consumption as a source of anthocyanins reported general improvement in some aspects of cognitive performance, including verbal memory and mood [27–29]. Another systematic review by Kent et al. [30] examining chronic consumption of anthocyanin-rich foods, such as blackcurrants, blueberries, cherries, and grapes, also suggested promising verbal memory benefits. In addition, in a critical review of epidemiological and randomized control studies in human subjects by Restani et al. [31] it was concluded that chronic consumption of 200–550 ml/day of red grape juice (for 30 days and more) significantly improved memory in the early stages of cognitive decline in older adults. However, Wang et al. [32] reported that fruit flavonoids did not have a significant role in improving cognition and mood. This seemingly conflicting evidence could be related to differences in sample sizes of studies included in the reviews and the different measurements used for cognitive domains examined in the meta-analyses. In addition, multiple functions can be tested within a single cognitive domain, using different tasks and tools. This adds further complexity to comparison between studies and further investigation is required.

Overall, while promising evidence is emerging regarding the protective role of anthocyanins in brain health, translation to dietary messaging and clinical practice is not yet possible due to variability in findings, and further well-designed studies are required to advance knowledge for this purpose. Nationally representative dietary survey data from Australia [33] suggests a mean intake of 24.17 mg/day (SE = 0.32) for habitual intake of anthocyanin, while a global composite database has estimated a slightly lower mean intake of 18.05 (SD 21.14) mg/day [34] for anthocyanin, showing that increase in usual anthocyanin intake across populations is possible. Similar previous systematic review and meta-analysis either measured the effect of all flavonoids or a specific food item on cognitive performance [29, 35–37] in a specific population [38]. To the best of our knowledge, this is the first inclusive systematic review and meta-analysis study to evaluate the effect of chronic administration of anthocyanins provided in different forms (i.e. food sources and supplements) and doses in randomized clinical trials on a variety of cognitive domains and mood, in the overall (cognitively healthy and unhealthy) adult population (> 18y).

## Methods and Materials

This systematic review and meta-analysis was conducted according to the Preferred Reporting Items for Systematic Reviews and Meta-analysis (PRISMA) guidelines [39] across all stages including data processing, analysis and reporting. The study protocol was registered in the Prospective Register of Systematic Reviews database (PROSPERO) [Registration code: CRD42021279470].

### Search Strategy

The systematic search was conducted using three databases (PubMed, Scopus, ISI Web of Science) as well as hand searching of the reference lists of identified relevant clinical trials published up to May 2024. The Population; Intervention; Comparator; Outcomes; Study type (PICOS) criteria for eligibility of studies are described in Table 1. Keywords and index terms used, based on the syntax rules of each database, are also provided [Supplementary material].

**Table 1:** PICOS criteria for inclusion of studies

Criteria	Description
Population	Adults aged > 18 yrs
Intervention	Anthocyanins provided as food or supplements (duration > 1wk)
Comparator	Control that did not contain anthocyanins
Outcomes	Cognitive domains, including memory, attention, executive function, speed of processing and mood
Study types	Randomized controlled clinical trials

### Eligibility Criteria

Titles and abstracts were screened, followed by the full text assessment of the eligible articles conducted in duplicate by two independent investigators (EL and VR).

*Inclusion criteria:* 1) Studies reporting the effects of anthocyanin intake on cognitive performance and mood in human randomised controlled trials (RCT) of either parallel or crossover design; 2) and studies published in the English language; 3) Studies that only captured sustained, longer-term impacts of anthocyanin on cognitive function and mood rather than immediate or transient effects of anthocyanins on outcomes (longer than 1 week).

*Exclusion criteria*: 1) One-group pre-test/post-test design; 2) studies that included patients with the following disorders: stroke, head injuries, severe mental disorders including disorders with psychotic symptoms such as schizophrenia, schizoaffective disorder, manic depressive disorder, Autism Spectrum Disorder, as well as severe forms of other mood disorders such as major depression, panic disorder, and obsessive–compulsive disorder according to DSM-5; 3) studies that investigated pregnant or breastfeeding participants; 4) participants under 18 years of age; and 5) studies that did not report doses of anthocyanin consumption. In the event of multiple publications resulting from the same trial, the publication that reported cognitive function outcomes was selected and included (Table 2).

**Table 2:** General characteristics of RCTs investigating the chronic interventions on cognition performance and mood in adults

Reference	Study design	Country	Sample size	Population details	Study duration	Anthocyanin Dose/source	Control	Cognitive tests	Mood outcomes	Results
Krikorian et al. 2012	Parallel	USA	21 Int: 10 Cont: 11	older adults with mild, age-related memory decline (68–90 yrs old)	16 weeks	425 mg/d concord grape juice (participants were give anthocyanin based on their weight)	Matched colour, taste, total calories, and sugar profile (placebo contained no juice or polyphenolic compounds)	CVLT	GDS	Reduced semantic interference on memory tasks (p = 0.04)
Small et al. 2014	Parallel	USA	105 Int: 52 Placebo: 53	Healthy participants (65–85 yrs old)	8 weeks	225mg/d/ NT-020: A pill consisted of 900 mg proprietary formulation of blueberry, carnosine, green tea, plus 200 U vitamin D3, 40 mg Biovin (Wild blueberry powder capsule)	A matched placebo pill	AVLT (episodic memory), Identical Pictures Test, the Number Comparison task (sustained attention tasks), and TMT A (processing speed), verbal ability, Forward and Backward DS task (working memory), TMT B, Category Fluency, and Controlled Oral Word Association (executive functioning), and Digit Symbol Test (complex speed)		Identical Pictures significant increases in performance (p = 0.021) and Number Comparison ( p = 0.012)
Lamport et al. 2016	Crossover	UK	25	healthy mothers (aged 40–50 yrs old) of preteen children	12 weeks	167 mg/d malvidin/ concord grape juice	Matched for energy, appearance, taste, volume, carbohydrate content, and all sugars	VVLT (verbal memory), VSLT (nonverbal spatial memory), RVIP (executive function), Grooved Pegboard (psychomotor skill), TOH (executive function)	VAS	Significant improvements in immediate spatial memory ( p < 0.05)
Kent et al. 2017	Parallel	Australia	42 Int: 21 Cont: 21	Mild-to-moderate dementia Alzheimer’s type in older adults (> 70 yrs oldrs)	12 weeks	69 mg/d /Cherry juice	Commercially prepared apple juice (degraded flavonoid contents)	RAVLT (verbal learning and memory), SOPT (working memory), BNT(semantic memory), TMT ( executive function), DSbackwards (short-term memory storage) and category letter verbal fluency (executive function)	GDS	Improvement in category verbal fluency task (p = 0.014), RAVLT total (p = 0.014), RAVLT delayed recall (p = 0.005) and RAVLT 20-min delayed recall (p ≤ 0.001)
Calapai 2017	Parallel	Italy	108 Int: 54 Cont: 54	Healthy older adults ( 56–74 yrs old)	12 weeks	> 32.5 mg/d/ Cognigrape capsule: Grape extract and maltodextrin	Placebo capsule: Maltodextrin	MMES, RBANS (assesses attention; language; visuospatial/constructional abilities; immediate memory; and delayed memory	Beck Depression Inventory (BDI); (self-report instrument that measures depression severity), Hamilton Anxiety Rating Scale (HARS); (evaluates anxiety through the investigation of 15 different areas (such as insomnia, mood, and somatic symptoms)	Mini-Mental State Examination baseline score was improved (p < 0.0001) decrease in BDI score (p < 0.0001), decrease in HARS (p < 0.05), improvements in (1) attention (p < 0.001); (2) language (p < 0.05); (3) immediate (p < 0.0001); and (4) delayed (p < 0.0001)
McNamara et al. 2017	Parallel	USA	39 Int: 19 Cont: 20	older adults with mild, self-perceived cognitive decline with aging ( 62- to 80yrs old)	24 weeks	269 mg/d/ freeze-dried blueberry powder	Matched for color, taste, and sugar content as (no polyphenolic contents)	DEX (working memory and executive function), TMT (psychomotor speed), Controlled Oral Word Production (lexical access), HVLT (learning and long-term memory)		Significant reduction (P = 0.05) in self-reported daily cognitive symptoms, Significant improvements (P = 0.04) in recognition memory on the HVLT
Bowtell et al. 2017	Parallel	UK	26 Int: 12 Cont: 14	healthy older adults (age 67.5 ± 3.0 y)	12 weeks	387 mg/d/ Blueberry concentrate	Synthetic blackcurrant and apple cordial with sugar added to match, identical to intervention in appearance (no polyphenol content)	the Groton maze timed chase test (speed of visual processing); the Groton maze learning test with a delayed recall component (executive function and delayed recall); identification task (attention); international shopping (verbal learning and delayed recall); and 1-back and 2-back memory tasks (working memory)		Improvement in working memory (2-back test) (p = 0.05)
Nilsson et al. 2017	Crossover	Sweden	40	healthy subjects ( 50–70 yrs old)	5 weeks (5 weeks washout)	414.2 mg/d/ Mixed berry beverage	Matched with respect to macronutrient composition, (insoluble and soluble), pH, polyphenols and antioxidant capacity	Selective Attention test Verbal Working Memory		Improved working memory (p = 0.05)
Bensalem et al. 2017	Parallel	Canada	190 Int: 92 Cont: 98	healthy older adults (60–70 yrs old)	24 weeks	33.54 mg/d/ polyphenol-rich extract from grape and blueberry	Maltodextrin providing no polyphenol	PALTEA (visuospatial learning and episodic memory test), VRMFR test (episodic verbal recall memory), VRMR test ( the verbal recognition memory immediate and delayed (20 min)), Spatial Span (SSP) and the Reverse SSP tests (working memory)		Significant improvement in immediate recall (p = 0.006),
Whyte 2018 et al	Parallel	UK	112 Int: 85 Cont: 27	healthy older adults (65–80 years)	24 weeks	11.35 mg/d/ wild blueberry powder and extract	The placebo powder (consisting of maltodextrin and food grade artificial dye—blue and red lake) was colour matched to make these treatments indistinguishable	RAVLT (verbal memory), visual episodic memory, Corsi Blocks task (visual memory span), serial subtractions and Sternberg memory scanning (Working Memory), MANT and Stroop task (executive function)	PANAS	Improvement in episodic memory performance p = 0.038,
Boespflug et al. 2018	Parallel	USA	16 Int: 8 Cont: 8	older adults with MCI (68 –92 years old)	16 weeks	269 mg/d/freeze-dried blueberry	Maltodextrin and food grade artificial dye—blue and red lake), colour matched	n-back (Working memory)		Not significant
Miller et al. 2018	Parallel	USA	38 Int: 19 Cont: 19	healthy older adults (60–75 years)	12 weeks	19.2 mg/g anthocyanin/ freeze-dried blueberry powder	Colour-matched, isocaloric, blueberry-flavoured placebo powder	Task switching (executive function). TMT (executive function and psychomotor speed), CVLT (Long-term memory), DS task ( Short-term memory), vMWM ( Spatial cognition), ANT (Attention)	GDS and the Profile of Mood States (POMS)	Significantly fewer repetition errors in the California Verbal Learning test (p = 0.03), reduced switch cost on a task-switching test (p = 0.03)
Joo et al. 2019	Parallel	South Korea	48 Int: 23 Cont: 25	participants with subjective memory impairment (aged 50 years or older)	12 weeks	19.08 mg/d/ cyanidin-3-glucoside-rich Oryza sativa L. (black rice) extract	Crystalline cellulose	ADAS-cog and CERAD-K (Azheimer’s assessment): VF (executive function), BNT (semantic memory), MMSE (cognitive impairment), word list (memory, recall, recognition) (Verbal memory), CP (Visuospatial memory), word list recall (WLR), CR (Visuospatial memory), TMT (executive function) , SMCQ (Subjective memory)		Significant improvement in subjective memory p = 0.043
Ahles et al. 2020	Parallel	Netherlands	101 Int: 69 Cont: 32	healthy adults ( 40–60 years)	24 weeks	43 mg/d/ Aronia melanocarpa extract (AME) black chokeberry	Maltodextrin	the Stroop colour and word test (executive function), the grooved pegboard test (psychomotor speed), and the number cross-out test (attention)	VAS	Improved psychomotor speed p = 0.009
Igwe et al. 2020	Crossover	Australia	28	generally healthy adults (adults aged 55 years and older)	8 weeks (4 weeks washout)	7.4–10.6 mg/d/ Queen garnet plum juice	Raspberry cordial (no polyphenol content)	RAVLT (verbal learning and memory), verbal fluency task (executive function), digit-span backwards task (short-term memory storage and executive function), Stroop task (executive function), and counting span (working memory)		Not significant
Krikorian et al. 2020	Parallel	USA	37 Int: 16 Cont: 21	sample of older adults with mild cognitive impairment ( aged 68 years and older)	16 weeks	258 mg/d/ freeze-dried blueberry powder	Artificial purple and red colouring, artificial blueberry flavour, natural blueberry flavour, maltodextrin, fructose, glucose, and citric acid	TMT (psychomotor speed), Controlled Oral Word Association (lexical and semantic access), HVLT (verbal memory), SPAL (visual-spatial learning), DEX (executive function)		Improved semantic access (p = 0.01), visual-spatial memory (p = 0.05),
Rosli et al. 2021	Parallel	Malaysia	31 Int: 16 Cont: 15	middle-aged women with signs of poor cognitive function ( aged 45–59 years)	10 weeks	194.1 mg/day/ tropical fruit juice	Formulated to contain no juice or natural polyphenol but looked and tasted similar	RAVLT (verbal learning and memory), DS (verbal working memory and short-term memory), CTMT(attention,)		significant interaction effects on RAVLT immediate recall (p < 0.05) and Comprehensive Trail Making Test (CTMT) Trail 4 (p < 0.05)
Kimble 2022 et al	Parallel	UK	50 Int: 25 Cont: 25	Healthy middle-aged adults (mean ± SD: 48 ± 6 years)	12 weeks	22.2 mg/d/ Montmorency cherry juice	Isoenergetic placebo	DV and RVIP (sustained attention and working memory) and N-back task (working memory)	Bond–Lader VAS	Significant improvement in sustained attention (higher DV accuracy p = 0.035, lower number of false alarms p = 0.005) Improvement in mood (higher alertness p = 0.013 and lower mental fatigue rating p = 0.009)
Flanagan et al. 2022	Parallel	UK	60 Int: 29 Cont: 30	Healthy older adults (50–80 years)	12 weeks	281 mg/d proanthocyanidins + 59 mg/d anthocyanin /Freeze-dried cranberry powder	Matched the active cranberry powder for taste	TMT: Executive functions and working memory, a short test of processing speed, attention, and set-shifting, and the Digit Span (DS) test, a subtest from the Weschler Adult Intelligence Scale–third edition (WAIS III) that assesses attention and short-term memory. RCF: short measure of visual memory and visuospatial constructional ability, The Supermarket Test: It includes a path integration test and measures (1) egocentric orientation, (2) short-term spatial memory, (3) heading direction, and (4) central (vs. boundary) based navigation preferences		Significant improvement in the intervention group in visual episodic memory (p = 0.026)
Cheatham et al. 2022	Parallel	USA	65 Int: 29 Cont:36	Adults experiencing mild cognitive decline ( 65–80 years)	24 weeks	411.25 mg/d anthocyanins + 284.9 mg/d Proanthocyanidins / a lyophilized wild blueberry powder	Matched placebo	*CANTAB MOT: Moto Screening Task RTI: Reaction Time SWM: Spatial Working Memory PAL: Paired Associate Learning		Not sig
Krikorian 2022 et al	Parallel	USA	27 Int: 13 Cont:14	Non-diabetic, middle-aged, overweight men and women with subjective cognitive decline ( 50–65 years)	12 weeks	140 mg/d/ Whole Freeze-Dried Blueberry	Matched for sugars, glycaemic load, appearance, and taste but did not contain fibre	Controlled Oral Word Association Test ( Executive ability), California Verbal Learning Test ( Learning/memory; Executive ability), Verbal Paired Associate Learning test ( Learning/memory), Everyday Memory Questionnaire ( Self-rated memory function)	BDI	Improved lexical access (p = 0.003), reduction of recall intrusion errors in verbal learning test ( p = 0.04),
Wood 2023 et al	Parallel	UK	54 Int: 27 Cont:27	Healthy older individuals (65–80 years)	12 weeks	302 mg/d/ Freeze-dried wild blueberry	Matched with appearance, taste, and macronutrient, fibre and vitamin C	AVLT (short-term verbal memory), Corsi Blocks (visualspatial memory), Serial 3’s and serial 7’s tasks (working memory), task-switching test (TST) (executive function, attention, and reaction time)	PANAS	Significant differences in immediate word recall (p = 0.04), improvement in accuracy score (p = 0.02)
Aarsland 2023 et al	Parallel	Norway	204 Int: 105 Cont: 99	Participants diagnosed with either mild cognitive impairment (MCI) or two or more cardiometabolic disorders (i.e., diabetes, hypertension, obesity) (60–80 years)	24 weeks	320 mg/d/ purified anthocyanin	Identically appearing placebo capsules (91% maltodextrin and 9% citric acid)	Modified Quality of Episodic Memory (Verbal memory) Attentional Intensity Index (Attention) Sustained Attention Index (Attention) Cognitive Reaction Time (Speed of processing) Attentional Fluctuation Index (Attention) Speed of Memory Retrieval Quality of Working Memory (working memory)		Not significant
Krikorian et al. 2023	Parallel	USA	30 Int: 15 Cont: 15	Overweight middle-aged men and women with insulin resistance and subjective cognitive decline (mean age 57 yrs)	12 weeks	36.8 mg/g/ whole-fruit strawberry powder	The placebo was designed to have the same appearance, taste, and carbohydrate load as the strawberry powder and contained fiber but no polyphenolic content	Trail-Making Test: working memory and set switching aspects of executive ability Controlled Oral Word Production: measure Lexical access, CVLT: assesses verbal learning and long-term memory function, SPAL: assesses ability to learn associations of visual-spatial stimuli	BDI	Sig reduction of interference in verbal learning and memory (p = 0.02) and lower mood disturbance ( p = 0.04)
Öz et al. 2024	Parallel	Turkey	39 Int: 20 Cont: 19	Participants diagnosed with mild to moderate Alzheimer’s disease (+ 65 years)	12 weeks	21.81 mg/d black mulberry (Morus nigra concentrate)	Nil	*MMSE (examining orientation, registration, attention, and calculation, recall and language), ADAS-Cog (word recall, naming (objects and fingers), following commands, constructions (drawing), ideational praxis, orientation, word recognition, recall of test instructions, spoken language ability, word finding difficulty and comprehension of spoken language, assessment of memory, praxis, and language	GDS	ADAS-Cog shows significant decrease in intervention group ( p = 0.002)
Curtis et al. 2024	Parallel	USA	20 Int: 9 Cont: 11	Participants with mild cognitive impairment (MCI) (mean age 76.33 ± 6.95)	24 weeks	15.9 mg/ American elderberry juice	placebo–control juice contained flavoured liquid with no nutritional content	MMSE: orientation to time, orientation to place, three-word registration, attention and calculation, three-word recall, language, and visual construction. HVLT: verbal learning and memory. BNT: measure of language. Rey CFT: visuospatial constructional ability and visual memory, Anagram problem solving tasks: verbal cognitive flexibility and convergent creativity, VPS: cognitive flexibility and convergent creativity		Not sig
P.Curtis et al. 2024	Parallel	USA	115 Int: 76 Cont: 39	Adults with metabolic syndrome (50–75 years)	24 week	364 Mg/d and 182 mg/d / freeze-dried blueberry powder	Isocaloric placebo	Attention (i.e., power of attention, cognitive reaction time, continuity of attention, and reaction time variability), Working Memory (i.e., quality of working memory), Episodic Memory (i.e., quality of episodic memory), Working and Episodic Memory (i.e., quality of memory), Speed of Retrieval from Memory (i.e., speed of memory), Executive Function, and Picture Recognition (i.e., original stimuli accuracy, new stimuli accuracy)	Bond-Lader VAS	Not sig
Gillies et al. 2024	Cross-over	New Zealand	20	Healthy females (18–45 years)	4 weeks (4 weeks washout)	151 mg/d /flavonoid-rich blackcurrant beverage (FBB)	taste-matched and appearance-matched control, also matched for macronutrient and vitamin C	The MTF (Purple Research Solutions): cognitive stressor, with mental arithmetic, Stroop, letter retrieval, and visual tracking tasks ( assessment of memory, psychomotor, and attentional performance)	STAI-S POMS	Letter retrieval ( p = 0.034) improved, mood (anger score) ( p = 0.043), tension scores ( p = 0.030) improved
Wattanathorn et al	Parallel	Thailand	69 Int: 46 Cont: 23	Healthy adults (45–65 years)	8 weeks	0.115 mg/d + 0.138 mg/d/ Anthaplex (purple waxy corn seed extract)	Matched placebo	Working memory: word presentation, word recognition test, picture presentation, picture recognition test, simple reaction test, digit vigilance, choice reaction time, spatial, and numeric working memory		Significant enhancement in power of attention and quality of memory
Velichkov et al. 2023	Parallel	UK	60 Int: 30 Cont: 30	Young adults with self-reported depression (18–24 years)	6 weeks	121 mg/d/ wild blueberry juice	blueberry-flavoured placebo drink matched for carbohydrates and fibre	modified version of a task-switching (executive function)	BDI-II, PANAS-X Generalized anxiety (GAD-7)	Reduction in BDI-II (p = 0.023) and GAD-7 (p = 0.026) in both intervention and control groups

*VPAL*: Verbal Paired associate learning test, *MMES*: Mini-Mental State Examination, *RBANS*: Repeatable Battery for the Assessment of Neuropsychological Status, *CVLT*: California Verbal Learning Test, *RAVLT*: Rey Auditory Verbal Learning Test, *SOPT*: Self-ordered pointing task, *TMT*: Trail Making Test, *BNT*: Boston naming test, *VVLT*: Visual Verbal Learning Test, *VSLT*: Visual Spatial Learning Test, *RVIP*: Rapid Visual Information Processing, *DEX*: The Dysexecutive Questionnaire, *HVLT*: Hopkin’s Verbal Learning Test, *PALTEA*: Paired Associate Learning total errors adjusted, *VRMFR*: *VRM* free recall, *VRMR*: VRM recognition, *MANT*: Modified Attention Network Test, *DS*: Digit Span, *vMWM*: Virtual Version of the Morris Water Maze, *ANT*: Attention Network Task, *CERAD-K*: Korean version of the Consortium to Establish a Registry for Alzheimer's Disease Assessment Packet, *ADAS-cog*: The Alzheimer's Disease Assessment Scale-Cognitive subscale, *SMCQ*: Subjective Memory Complaints Questionnaire, *VF*: Verbal Fluency, *MMSE*: the Mini Mental Status Examination, *WLM*: Word List Memory, *CP*: Constructional Praxis, *CR*: Constructional Recall, *SPAL*: Spatial Paired Associate Learning Test, *CTMT*: Comprehensive Trail Making Test, *VAS*: visual analogue scale, *DV*: digit vigilance, *BDI*: Beck Depression Inventory, *GDS*: Geriatric Depression Scale, *POMS*: the Profile of Mood States, *HARS*: Hamilton Anxiety Rating Scale, Rey *CFT*: Rey–Osterrieth Complex Figure Test, *VPS*: Visuospatial Problem Solving, Cantab: Cambridge neurological test automated battery, *STAI-S*: State Trait Anxiety Inventory—State Portion,

### Data Extraction

The general information extracted from each study included: Author’s last name, publication year, country, baseline characteristics of the study population (sample size, age, and health status), study design (crossover or parallel), intervention duration, anthocyanin dose (mg/day), type of control, cognitive and mood tests, and the main study findings. In the case of missing data, attempts were made to contact the authors via email. Data extraction was performed by one author (EL), with oversight and discussion by KC and KW-G.

### Quality Assessment

The revised version of Cochrane risk of bias tool (RoB2) [40, 41] was used to evaluate the quality of each study according to the following five criteria parameters: I) randomization process; II) effect of assignment to intervention; III) missing outcome data; IV) measurement of the outcome and V) selection of the reported result. Each domain consists of a series of questions that can form a judgement of “low” or “high” risk of bias or can express “some concerns”. Each study was then assigned an overall risk of bias, as follows: Low risk of bias (low allocated for all domains); Some concerns (some concerns in at least one domain, but not to be at high risk of bias for any domain) and high risk (high in at least one domain or some concerns for multiple domains). The quality rating assessment of studies was performed by EL in discussion with KC, KW-G and SR. All eligible studies were summarised as a qualitative synthesis before meta-analysis. Risk-of-bias VISualization (robvis) was applied to summarize the risk of bias assessment [42]

## Statistical Analysis

Comprehensive Meta-Analysis (CMA) software [43] was used for quantitative analysis. For intervention and control groups, within-group changes in outcomes from baseline to post-intervention and their changed standard deviations (SDs) were extracted from each study. If the studies did not report the change values, the baseline and final mean values and their standard deviations (SDs) were extracted, and the SD of mean changes was calculated using correlation coefficient of 0.5. The meta-analyses were also conducted using *r* = 0.2 and *r* = 0.8 to determine if the overall effects were sensitive to the selected correlation coefficient.

Effect sizes (Hedge’s g score) were calculated to account for the various methods utilised across the studies, correct for bias in studies with small sample sizes, and to provide a common unit of measurement between studies as cognition outcomes varied widely, both in terms of the instruments used but also according to different units for the various measurements.

The overall Hedge’s g and corresponding confidence limits were then derived by taking variability into account using a random-effects model. To calculate the total percentage of variation described as between-study heterogeneity, Cochrane’s Q test and I-squared (I2) statistics were applied, with the values of 0–25%, 26–75%, or 76–100% indicating a low, moderate, or high degree of heterogeneity, respectively [44]. To assess the overall robustness of the results, sensitivity analyses were planned [45]. Egger’s regression asymmetry test and Begg’s adjusted rank correlation test, along with visual inspection of the funnel plots, were conducted to investigate any publication bias [46]. The included randomized controlled trials (RCTs) employed an extensive array of neuropsychological tests categorized into distinct neurocognitive domains. This classification relied on data supplied by the authors and systematic reviews published earlier [38, 47].

## Results

### Study Selection

**Fig. 1:**
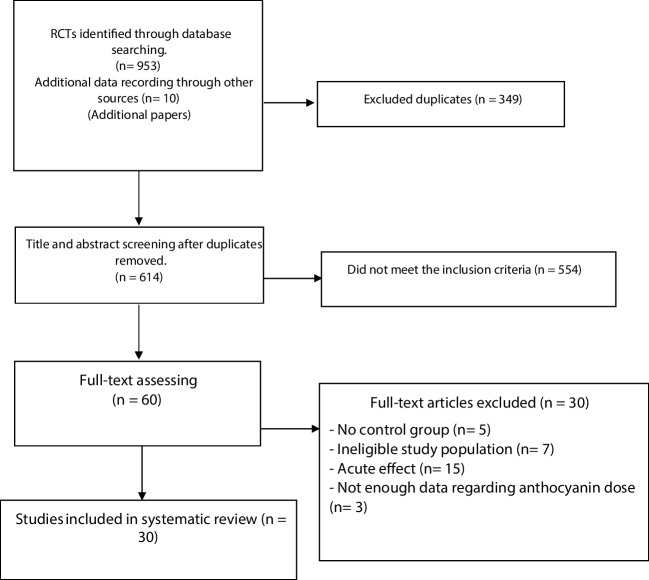
PRISMA Flow Diagram for systematic research and identification of studies meeting the inclusion criteria

The PRISMA flow diagram [48] (Fig. 1) shows that 953 randomized clinical trials were detected through the systematic search of the three databases. An additional 10 studies were identified through manual checking of reference lists. Of those, 349 studies were identified as duplicates and excluded. While screening the titles and abstracts of the studies identified through the initial search phase, 554 studies that did not meet the inclusion criteria were removed. Following a full-text assessment of 60 studies for eligibility, five studies were excluded because of a lack of control group [49–53] seven due to an ineligible study population [54–60], 15 because of intervention duration of one week and less [61–75], while three studies did not provide data regarding anthocyanin dose [76–78]. Authors were contacted several times via email in an attempt to obtain missing data regarding dose of anthocyanins, but no response was forthcoming. As a result, 30 studies were included in the final review. Of these, 14 (n = 733 participants) reported mean and standard deviation values and were therefore eligible for meta-analysis [79–92].

### Characteristics of the Included Trials

The general characteristics of the studies that met the inclusion criteria are presented in Table 2. Studies included in this systematic literature review and meta-analysis were published between 2012 and 2024, with 26 studies employing a parallel study design [79, 81, 83–106], while the remaining four were cross-over studies [80, 82, 107, 108]. Eleven studies were conducted in the United States [87–89, 92–94, 97, 99, 101, 102, 104], seven in the United Kingdom [82, 85, 90, 91, 95, 105, 109], two in Australia [80, 81] and one each in New Zealand [108], South Korea [83], Malaysia [84], Canada [86], Sweden [107], Turkey [103], Norway [98], Italy [100], Netherlands [96], and Thailand [106]. The duration of treatment ranged from 4 to 24 weeks. Eleven studies investigated anthocyanin effects amongst a population of older adults (62–92 y) with mild to moderate cognitive impairment and/or memory conditions [81, 83, 87, 92–94, 97–99, 101, 103]. Two studies included middle-aged adults with mild cognitive impairment [84, 102], while 15 studies were conducted in cognitively healthy middle aged and older adults (40–85 y) [79, 80, 82, 85, 86, 88–91, 95, 96, 100, 104, 106, 107]. One study included healthy young women (18–45 y) [108] and another healthy young adults with self-reported depression (18–24 y) [105]. Anthocyanins provided in the included studies were derived from various sources, such as Aronica melano carpa (black chokeberry) extract [96], blueberry (or blueberry freeze-dried/powder) [79, 87–90, 94, 95, 97, 99, 101, 104, 105], Queen Garnet plum [80], black rice extract [83], cherry juice [81, 110], concord juice [82, 93], grape extract capsule [100], tropical fruit juice [84], mixed berries [107], a mixture of grape and blueberry [86], purple waxy corn seed extract (Anthaplex) [106], blackcurrant beverage [108], American elderberry [92], black mulberry (Marus Nigra concentrate) [103], whole fruit strawberry powder [102] and freeze-dried cranberry powder [91]. One study used purified anthocyanin capsules as the intervention [98]. Anthocyanin doses provided in the studies varied between 10 mg [80] and 425 mg/day [93]. Most studies noted that placebo products used as control treatments were similar in texture, taste, and colour as the intervention. However, some studies used apple juice [81], blackcurrant and apple cordial (which was matched in appearance and taste with intervention juice) [79] or raspberry cordial [80] for the control which contained zero or small amount of polyphenolics. One study did not provide a placebo to control participants [103]. Neurological tests were categorized into their respective cognitive domains for the purpose of the meta-analysis as follows: verbal learning [79–84, 89, 90, 92], immediate memory [80, 81, 86, 88], delayed recall memory [79–83, 88–90, 92, 111] and working memory [80, 81, 84, 85, 87, 89, 91].

### Risk of Bias Assessment

**Fig. 2:**
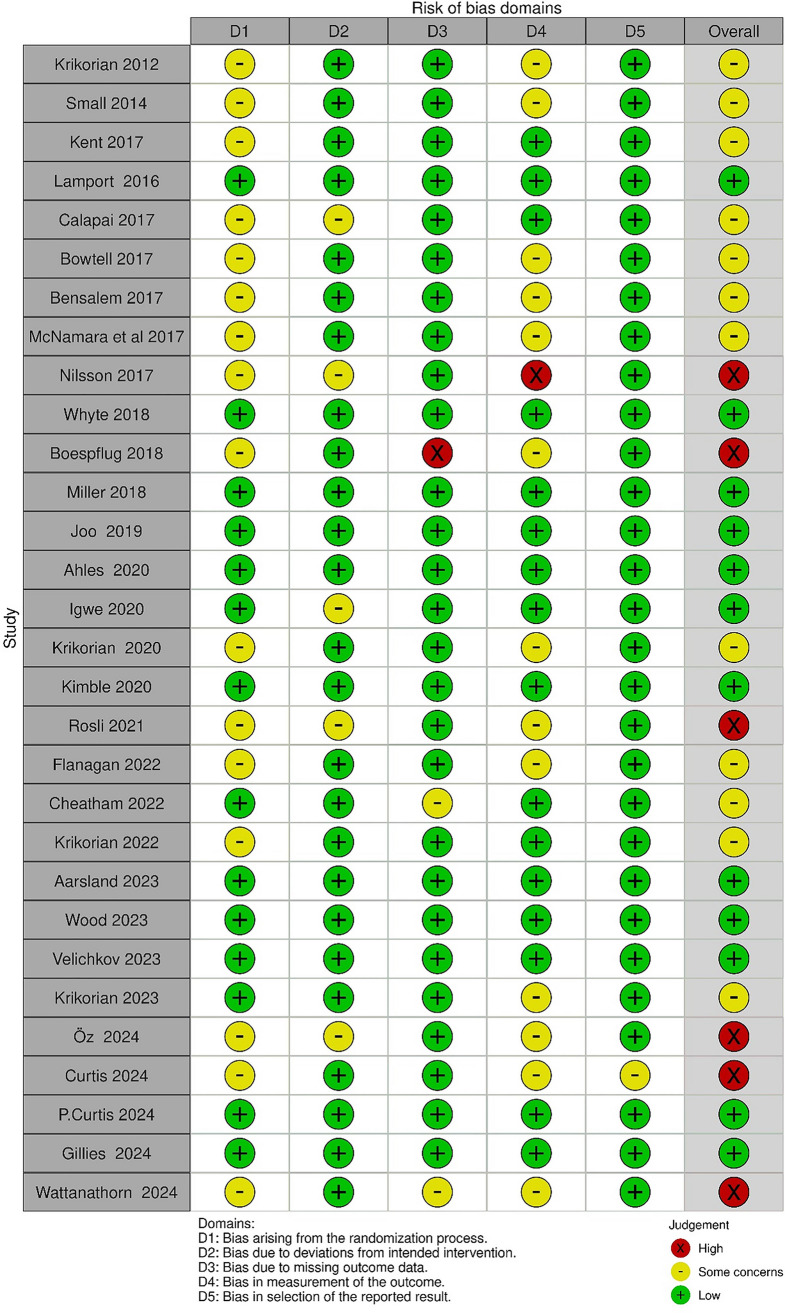
RoB2 summary and graph

As shown in Fig. 2, the RoB2 (Risk of Bias) assessment results are presented using both a traffic light plot and as percentage breakdowns. For half of the studies (53.3%), there were “some concerns” regarding the randomization process [79, 81, 84, 86, 87, 89, 92–94, 97, 99, 100, 103, 106, 107, 112], while the remaining studies had “low” risk of bias for this domain. For the domain of assessing the effect of assignment to intervention, only five studies (16.1%) raised “some concerns” [80, 84, 103, 107, 113], with the remainder judged as having a “low” risk of bias.

For the missing outcome data domain, two studies raised “some concerns” [101, 106], while one study was rated as “high” risk of bias [87]. The outcome measurement domain presented a “high” risk of bias for one study (Nilsson et al. [107]) and “some concerns” for 13 studies (43.33%) [79, 84, 86, 87, 89, 92–94, 97, 102, 103, 106, 112]. For the final domain (selection of reported results), most studies were evaluated as having a “low” risk of bias, with only one study rated as having “some concerns” [92].

In terms of overall risk of bias, approximately 40% of studies had “low” risk of bias while 20% exhibited a “high” risk of bias due to either one high-risk rating or multiple areas of concern. The remaining studies (40%) demonstrated “some concerns”. The comprehensive RoB2 evaluation details are provided in Table 3.

**Table 3:** Risk of bias assessment of studies included in the review and meta-analysis utilising the Cochrane Collaboration Risk of Bias Tool (RoB2)

Study	1.1	1.2	1.3	Risk-of-bias judgement for randomization process	2.1	2.2	2.3	2.4	2.5	2.6	2.7	Risk-of-bias judgement for effect of assignment to intervention)	3.1	3.2
Krikorian 2012	Yes	NI	No	Some concerns	No	No	NA	NA	NA	PY	NA	Low	Yes	NA
Small 2014	Yes	NI	PN	Some concerns	No	No	NA	NA	NA	Yes	NA	Low	No	Yes
Kent 2017	Yes	NI	No	Some concerns	No	PN	NA	NA	NA	NA	NA	Low	No	Yes
Lamport 2016	Yes	Yes	No	Low	No	NA	NA	NA	NA	NA	NA	Low	Yes	NA
Calapai 2017	Yes	NI	No	Some concerns	No	NI	NI	NA	NA	Yes	NA	Some concerns	No	Yes
Bowtell 2017	NI	NI	PN	Some concerns	No	No	NA	NA	NA	NA	NA	Low	Yes	NA
Bensalem 2017	NI	NI	PN	Some concerns	No	No	NA	NA	NA	NA	NA	Low	No	Yes
McNamara et al. 2017	NI	Ni	PN	Some concerns	No	No	NA	NA	NA	PY	NA	Low	No	No
Nilsson 2017	Yes	NI	NI	Some concerns	Yes	NI	NA	NA	NA	NA	NA	Some concerns	No	Yes
Whyte 2018	Yes	PY	No	Low	No	No	NA	NA	NA	NA	NA	Low	No	Yes
Boespflug 2018	NI	NI	No	Some concerns	No	No	NA	NA	NA	NA	NA	Low	No	PN
Miller 2018	Yes	PY	No	Low	No	No	NA	NA	NA	NA	NA	Low	No	Yes
Joo 2019	Yes	PY	No	Low	No	No	NA	NA	NA	NA	NA	Low	Yes	NA
Ahles 2020	Yes	PY	No	Low	No	No	NA	NA	NA	PY	NA	Low	Yes	NA
Igwe 2020	Yes	PY	No	Low	PY	No	NI	NA	NA	NI	PN	Some concerns	No	Yes
Krikorian 2020	NI	NI	NI	Some concerns	No	No	NA	NA	NA	NA	NA	Low	No	Yes
Kimble 2020	Yes	PY	No	Low	No	No	NA	NA	NA	Yes	NA	Low	No	Yes
Rosli 2021	Yes	NI	No	Some concerns	PN	NI	NI	NA	NA	NA	NA	Some concerns	Yes	NA
Flanagan 2022	NI	NI	No	Some concerns	No	No	NA	NA	NA	NA	NA	Low	Yes	NA
Cheatham 2022	Yes	PY	No	Low	No	No	NA	NA	NA	NA	NA	Low	No	PN
Krikorian 2022	NI	NI	No	Some concerns	No	No	NA	NA	NA	NA	NA	Low	No	Yes
Aarsland 2023	Yes	PY	No	Low	No	No	NA	NA	NA	NA	NA	Low	Yes	NA
Wood 2023	Yes	Yes	No	Low	No	No	NA	NA	NA	NA	NA	Low	Yes	NA
Velichkov 2023	Yes	Yes	No	Low	No	No	NA	NA	NA	NA	NA	Low	Yes	NA
Krikorian 2023	Yes	PY	No	Low	No	No	NA	NA	NA	NA	NA	Low	Yes	NA
Öz 2024	Yes	NI	No	Some concerns	NI	NI	NI	NA	NA	NI	PN	Some concerns	No	PN
Curtis 2024	NI	NI	PN	Some concerns	No	No	NA	NA	NA	NA	NA	Low	No	Yes
P.Curtis 2024	Yes	PY	No	Low	No	No	NA	NA	NA	PY	NA	Low	No	Yes
Gillies 2024	Yes	PY	No	Low	No	No	NA	NA	NA	PY	NA	Low	Yes	NA
Wattanathorn 2024	NI	NI	No	Some concerns	No	No	NA	NA	NA	NA	NA	Low	No	NI

Study	3.3	3.4	Risk-of-bias judgement for missing outcome data	4.1	4.2	4.3	4.4	4.5	Risk-of-bias judgement for measurement of the outcome	5.1	5.2	5.3	Risk-of-bias judgement for selection of the reported result	**Overall risk of bias**
Krikorian 2012	NA	NA	Low	No	No	NI	PY	PN	Some concerns	Yes	PN	PN	Low	Some concerns
Small 2014	NA	NA	Low	No	PN	NI	NI	PN	Some concerns	Yes	PN	PN	Low	Some concerns
Kent 2017	NA	NA	Low	No	No	No	NA	NA	Low	Yes	No	No	Low	Some concerns
Lamport 2016	NA	NA	Low	No	No	PN	NA	NA	Low	Yes	No	PN	Low	Low
Calapai 2017	NA	NA	Low	No	No	NI	Yes	PN	Low	Yes	No	No	Low	Some concerns
Bowtell 2017	NA	NA	Low	No	PN	NI	PY	PN	Some concerns	Yes	PN	PN	Low	Some concerns
Bensalem 2017	NA	NA	Low	No	No	NI	Yes	No	Some concerns	Yes	PN	PN	Low	Some concerns
McNamara et al. 2017	No	NA	Low	PN	No	NI	Yes	PN	Some concerns	Yes	No	PN	Low	Some concerns
Nilsson 2017	NA	NA	Low	No	No	NI	PY	PY	High	Yes	No	No	Low	High
Whyte 2018	NA	NA	Low	No	No	Yes	NA	NA	Low	Yes	No	No	Low	Low
Boespflug 2018	PY	NI	High	PN	No	NI	NI	PN	Some concerns	Yes	PN	PN	Low	High
Miller 2018	NA	NA	Low	No	No	No	NA	NA	Low	Yes	No	No	Low	Low
Joo 2019	NA	NA	Low	No	No	No	NA	NA	Low	Yes	No	No	Low	Low
Ahles 2020	NA	NA	Low	PN	No	No	NA	NA	Low	Yes	PN	No	Low	Low
Igwe 2020	NA	NA	Low	No	No	PN	NA	NA	Low	Yes	No	No	Low	Low
Krikorian 2020	NA	NA	Low	No	No	NI	NI	PN	Some concerns	Yes	No	No	Low	Some concerns
Kimble 2020	NA	NA	Low	No	No	PN	NA	NA	Low	Yes	No	No	Low	Low
Rosli 2021	NA	NA	Low	PN	No	NI	PY	PN	Some concerns	Yes	No	No	Low	High
Flanagan 2022	NA	NA	Low	No	PN	NI	NI	PN	Some concerns	Yes	PN	PN	Low	Some concerns
Cheatham 2022	PY	PN	Some concerns	No	No	No	NA	NA	Low	Yes	No	No	Low	Some concerns
Krikorian 2022	NA	NA	Low	No	No	PN	NA	NA	Low	Yes	No	No	Low	Some concerns
Aarsland 2023	NA	NA	Low	No	No	No	NA	NA	Low	Yes	No	No	Low	Low
Wood 2023	NA	NA	Low	No	No	No	NA	NA	Low	Yes	No	No	Low	Low
Velichkov 2023	NA	NA	Low	No	No	No	NA	NA	Low	Yes	No	No	Low	Low
Krikorian 2023	NA	NA	Low	No	No	NI	PY	PN	Some concerns	Yes	PN	No	Low	Some concerns
Öz 2024	PN	NA	Low	PN	No	NI	PY	PN	Some concerns	Yes	PN	PN	Low	High
Curtis 2024	NA	NA	Low	No	No	NI	Yes	PN	Some concerns	NI	NI	NI	Some concerns	High
P.Curtis 2024	NA	NA	Low	No	No	No	NA	NA	Low	Yes	PN	PN	Low	Low
Gillies 2024	NA	NA	Low	No	No	No	NA	NA	Low	Yes	No	No	Low	Low
Wattanathorn 2024	NI	PN	Some concerns	No	PN	NI	PY	PN	Some concerns	Yes	PN	PN	Low	High

### Qualitative Synthesis

Based on the information presented in Table 2, the cognitive domains were assessed by these studies using diverse tasks and methods of measurement. However, despite the variation across studies, improvement was observed in at least one cognitive task in some of the studies following anthocyanin administration [86, 88–91, 93–95, 97, 99, 102, 103, 106–108]. Additionally, a few studies reported enhanced mood outcomes post-anthocyanin supplementation. [85, 100, 102, 108].

#### Verbal Learning and Memory

Sixteen studies assessed verbal learning and memory, of which 10 found significant improvements using tools such as the Rey Auditory Verbal Learning Test (RAVLT) [81, 84, 90, 95], the Hopkins Verbal Learning Test [94], California Verbal Learning Test (CVLT) [88, 93, 99, 102] and Repeatable Battery for the Assessment of Neuropsychological Status (RBANS) (immediate and delayed memory test) [100]. These tools require participants to learn a list of words over multiple repeated presentations. Studies that did not report significant benefits used the following tests to measure verbal learning and memory: Word List (memory, recall and recognition) [83], international shopping list task [79], Visual Verbal Learning test (VVLT) [82], Hopkins Verbal Learning Test [92, 97], RAVLT [80] and Quality of Episodic Memory [98]. Studies that reported significant findings in verbal learning and memory used doses of anthocyanin ranging from 11.35 mg/day to 302 mg/day. Whyte et al. [95] provided 11.35 mg/day of anthocyanins from blueberry extract powder in a population of healthy older adults, while Kent et al. [81] provided 69 mg/d via cherry juice to cognitively impaired older adults. Two studies in adults with subjective cognitive decline provided 140 mg/d from whole freeze-dried blueberries capsules [99] and 36.8 mg/d anthocyanin from whole-fruit strawberry powder [102] respectively. Another investigation in adults with mild to moderate cognitive impairment provided 194.1 mg/d from tropical fruit juice [84]. The highest dose study was provided to healthy older adults with 302 mg/d anthocyanins from freeze dried blueberry powder [90].

#### Executive Function

Executive function, an umbrella term for high-order cognitive processes involving the frontal lobes, includes functions such as task switching and attentional control and working memory. Various executive functions were evaluated with significant improvements reported in four studies. Firstly, task switching was improved in studies that used 19.2 [88] mg/d and 320 mg/d [90] anthocyanin from blueberry powder respectively in a healthy population; verbal fluency (p = 0.014) [81] improved following 69 mg/d anthocyanin from cherry juice over 12 weeks; and subjective reporting of multiple capacities in the Dysexecutive Questionnaire (p = 0.05) was improved with 269 mg/d anthocyanin from blueberry powder in older adults with mild, self-perceived cognitive decline with aging [94]. Other studies assessing this cognitive domain did not report significant findings utilising the following tests: Trail Making Test (with anthocyanin doses of 69 mg/d from cherry juice [81], 19.08 mg/d from black rice extract [83], 19.2 mg/d from freeze-dried blueberry powder [88], 281 mg/d proanthocyanidin + 59 mg/d anthocyanin from freeze-dried cranberry powder [91] and 36.8 mg/d from whole-fruit strawberry powder [102]); Rapid visual information processing (RVIP) and Tower of Hanoi (167 mg/d anthocyanin intake from concord grape juice) [82]; the Groton maze learning test (387 mg/d anthocyanin from blueberry concentrate) [79]; the Modified Attention Network Task (MANT) (11.35 mg/d anthocyanin from wild blueberry powder and extract) [95]; Stroop task (with anthocyanin doses of 7.4–10.6 mg from Queen Garnet plum juice [80], 43 mg/d from Aronia melanocarpa extract (AME) black chokeberry [96], 11.5 mg/d from wild blueberry powder and extract [95]; verbal fluency task (with anthocyanin doses of 7.4–10.6 mg/d from Queen Garnet plum, 19.08 mg/d from black rice extract, and 225 mg/d anthocyanin from blueberry capsules, respectively [80, 83, 89]); digit span backwards (with anthocyanin doses of 7.4–10.6 mg/d from Queen Garnet plum) [80]; DEX (258 mg/d anthocyanin from freeze-dried blueberry powder) [97]; task switching test (121 mg/d anthocyanin from blueberry juice [105]).

#### Working Memory

Working memory was examined in 18 studies, with improvement observed on a sentence span task by Nilsson et al. [107] (*p* = 0.05) after 5 weeks of treatment with a mixed berry beverage (414.2 mg/d anthocyanin) in healthy subjects, and Bowtell et al. [94] in 1-back and 2-back memory tasks (*p* = 0.05) with 387 mg/d anthocyanin from blueberry concentrate in healthy adults after 12 weeks of treatment. On the contrary, the rest of the studies reported no effect of anthocyanin administration via blueberry and grape juice, blueberry freeze-dried powder, Queen Garnet plum juice, tropical fruit juice or, Montemorency cherry juice fruit, purple waxy corn seed extract, whole-fruit strawberry powder, freeze-dried cranberry powder and purified anthocyanin capsules. These studies utilised a variety of tasks to evaluate working memory with various doses of anthocyanin intake: Spatial Span (SSP) and the Reverse SSP tests (33.54 mg/d anthocyanin) [86]; serial subtractions (302 mg/d anthocyanin) and Sternberg memory scanning (11.35 mg/d) [90, 95]; n-back (269 mg.d) [87]; counting span (10.6 mg/d) [80]; Digit Span (194.1 mg/d and 225 mg/d) [84, 89]; rapid visual information processing (RVIP) (22.2 mg/d) [85]; the Self-ordered pointing task (SOPT) (69 mg/d) [81]; and quality of working memory (320 mg/d) [98]and (364 mg/d) [104], trail making test ( 281 mg/d proanthocyanidins + 59 mg/d anthocyanin and 36.8 mg/g)[91, 102], spatial working memory (411.25 mg/d anthocyanins + 284.9 mg/d Proanthocyanidins) [101] and numeric working memory [106]

#### Psychomotor Speed

To measure psychomotor speed, the grooved pegboard was used in two studies [82, 96]; however, only Ahles et al. [96] observed improvements in the intervention group compared to control (p = 0.009) using 43 mg/d anthocyanin from Aronia melanocarpa extract (AME) black chokeberry as an intervention. Psychomotor speed was also measured in other studies through tools such as the Trail-Making Test (269 mg/d [94], 268 mg/d [97], 19.2 mg/d [88] anthocyanin from freeze-dried blueberry), digit vigilance (268 mg/d anthocyanin from freeze-dried blueberry and 320 mg/d purified anthocyanin capsules) [97, 98] and visual tracking tasks (151 mg/d from flavonoid-rich blackcurrant beverage) [108], however, no significant change was observed.

#### Attention

Attention improved in studies that used rapid visual information processing (RVIP) and Digit Vigilance (DV; higher DV accuracy *p* = 0.035, lower number of false alarms *p* = 0.005; 194.1 mg/day anthocyanin from tropical fruit juice) [85] and numeric working memory (significantly increased in the power of attention in % of accuracy *p* = 0.015 with 0.115 mg/d anthocyanin and *p* = 0.046 with 0.138 mg/d anthocyanin from purple waxy corn seed extract (Anthaplex)) [106], Comprehensive Trail Making Test (CTMT) (*p* < 0.05; 22.2 mg/d anthocyanin from Montmorency cherry juice) [111] and identical pictures and number comparison tasks (225 mg/d anthocyanin from wild blueberry powder) [89]. The RBANS test was also used by Calapai et al. [100] to assess a series of cognitive domains, including attention, which resulted in significant benefits (*p* < 0.001) following administration of > 32 mg/d anthocyanins from grape extract. Additionally, other studies failed to find an effect using an identification task (387 mg/d from blueberry concentrate) [79], Selective Attention (SA) test (414.2 mg/d from mixed berry beverage) [107], Attention Network Task (ANT) (19.2 mg/g anthocyanin from freeze-dried blueberry powder) [88], the number cross-out test (43 mg/d anthocyanin from Aronia melanocarpa extract (AME) black chokeberry) [96], attention measured using through three indicators (Attentional Intensity Index, Sustained Attention Index and Attentional Fluctuation Index) (302 mg/d purified anthocyanin) [98], visual tracking tasks (assessing attentional performance with 151 mg/d anthocyanin from flavonoid-rich blackcurrant beverage) [108], power of attention (364 mg/d mg from 1 cup of freeze-dried blueberry and 182 mg/d anthocyanin from ½ cup of freeze-dried blueberry) [104], two studies performed Mini-Mental Status Examination (MMSE) measurement assessing attention and calculation (using 15.9 mg/d anthocyanin from American elderberry juice and 21.81 mg/d from black mulberry (Morus nigra concentrate), attention measurement through trail making test and digit span as a subtext of Weschler Adult Intelligence Scale–third edition (WAIS III) [91].

#### Other Cognitive Domains

Few studies have examined performance in other cognitive domains or tasks. For example, short-term memory was assessed by Digit Span in only five studies [80, 81, 84, 88, 91] and Corsi Block test in one study [90], all of which found no significant effect. Six studies measured Visual Spatial function, however only two reported significant improvements after anthocyanin intake using the Visual Spatial Learning Test (VSLT) (*p* < 0.05) via concord grape juice (167 mg/d malvidin)[82], the Spatial Paired Associate Learning Test (SPAL) (*p* = 0.05) via freeze-dried blueberry powder (258 mg/d anthocyanin) [97]. The tools for measurement differed in the four studies that did not report significant findings, and included: Paired Associated Learning total errors adjusted (PALTEA) (33.54 mg/d anthocyanin from polyphenol-rich extract of grape and blueberry) [86]; a virtual version of the Morris Water Maze (vMWM) (19.2 mg/d anthocyanin from freeze-dried blueberry powder) [88]; Constructional Praxis/recall (19.08 mg/d anthocyanin from Oryza sativa L. (black rice) extract) [83] and Repeatable Battery for the Assessment of Neuropsychological Status (RBANS) test (< 32.5 mg/d anthocyanin from grape extract) [100]. Only two studies measured semantic memory using the Boston naming test (69 mg/d anthocyanin from cherry juice and 19.08 mg/d cyanidin-3-glucoside from Oryza sativa L. (black rice) extract) [81, 83] while another used the Controlled Oral Word Association (258 mg/d anthocyanin from freeze-dried blueberry powder) [97]. Of these, only the Krikorian et al. study [97] found significant effects. Significant improvements in visual episodic memory was observed (*p* = 0.026) in one study (281 mg/d proanthocyanidins + 59 mg/d anthocyanin from freeze-dried cranberry powder for 12 weeks) [91].

#### Mood

Mood was evaluated by various tests across 15 studies, including the Geriatric Depression Scale (GDS) [81, 88, 93, 103], Visual Analogue Scales (VAS) [82, 96, 104], Bond-Lader VAS [110], the Profile of Mood States (POMS) [88, 108], the Positive and Negative Affect Schedule (PANAS) [90, 95, 105], the Beck Depression Inventory (BDI) [99, 100, 102, 105] which is a self-report instrument that measures depression severity, and Hamilton Anxiety Rating Scale (HARS) that evaluates anxiety through the investigation of 15 different areas such as insomnia, mood, and somatic symptoms [100]. Significant findings were observed in the study by Kimble et al. (22.2 mg/d anthocyanin from Montmorency cherry juice) [110] which showed higher alertness using the Bond-Lader VAS (*p* = 0.013), accompanied by a decrease in fatigue (*p* = 0.009) in the intervention group. Furthermore improvements in anger score (*p* = 0.04) and tension score (*p* = 0.03 was observed in a study by Gillies et al. [108] following 151 mg/d anthocyanin consumption in the form of a blackcurrant beverage. Additionally, a significant decrease in BDI score (*p* < 0.05) was reported in multiple studies [100, 102, 105] following consumption of Cognigrape (> 0.32 mg/d anthocyanin from grape extract) capsule consumption for 12 weeks, 36.8 mg/d anthocyanin from strawberry powder for 12 weeks and 121 mg/d from wild blueberry juice for 6 weeks, respectively, however the Velichkov et al. study reported a significant reduction in BDI score for both treatment and placebo groups, with a greater decrease in the placebo group.

#### Challenges in Comparing Cognitive Outcomes Across Studies

As previously mentioned, different instruments we often used to measure the same cognitive domain between studies which makes comparison difficult. In studies that used the same tasks to assess a cognitive function, differences in the outcome could be related to factors such as variable sample size, anthocyanin dose, participant characteristics or the duration of the study. For example, both Boespflug et al. [87] and Bowtell et al. [79] used blueberry as the main source of anthocyanin; however, Boespflug et al. [87] administered 269 mg/d anthocyanins as freeze-dried powder in older participants with mild cognitive impairment (*n* = 16) and did not observe an improvement in working memory, while Bowtell et al. [79] reported increased working memory associated with a dose of 387 mg/d anthocyanins provided as a blueberry concentrate in healthy adults (*n* = 26).

The domain that appeared to be the most consistently researched was verbal learning memory, whereby most studies using a verbal learning task reported a significant effect. Many of these studies utilised similar tasks. Three studies that used the Hopkins verbal learning test reported contrasting results; significant positive findings were reported by McNamara et al. [94] whereas Krikorian et al. [97] and Curtis et al. [92] found no effect of anthocyanins on test scores. Both the McNamara et al. and Kriokorian et al. studies used a similar dosage of freeze-dried blueberry powder as the source of anthocyanin in a relatively small sample size (*n* = 39 and 37, respectively); however, the intervention continued for 24 weeks in McNamara et al. [94], compared to 16 weeks in the Krikorian et al. [97] study. The Curtis et al. [92] (*n* = 20) study provided participants with mild cognitive impairment a lower dose of anthocyanin (15.9 mg/d) provided by American elderberry administered for 24 weeks. Overall, these results indicate significant cognitive improvements, particularly among participants who were relatively cognitively healthy. [94]. Furthermore, Igwe et al. [80] reported no significant benefit of low-dose anthocyanin supplementation using the RAVLT as the instrument to measure verbal memory (*n* = 28 participants).

Contrasting findings were also observed across studies that examined executive function. For example, the DEX was used by McNamara et al. [94] to measure both subjective working memory and executive function, with significant improvements reported for the executive function component of the DEX after anthocyanin intervention, but not working memory. The same test was utilised by Krikorian et al. [97] to measure executive function; however, no significant results were observed. Additionally, Miller et al. [88] applied two different objective tests (Trail Making Test and Task Switching) to evaluate executive function; however, only task switching showed significant benefits of anthocyanin supplementation. This is in line with another study that utilised task switching to measure executive function and found significant positive results [90]. However, Kent et al. [81] who also used the trail making test and verbal fluency to measure executive function reported improvements only for verbal fluency. On the contrary, verbal fluency was also measured by Igwe et al. [80] and Joo et al. [83], with both studies reporting no significant effects of anthocyanins on executive function; however, Joo et al. [83] reported improved subjective memory (assessed by Subjective Memory Complaints Questionnaire) after anthocyanin intake. Psychomotor speed was significantly improved by anthocyanins measured using the grooved peg-board in Ahles et al. [96], but these improvements were not apparent in a study by Lamport et al. [82]. This may be explained by differences in samples size (*n* = 101 compared to *n* = 19, respectively) and longer duration (24 vs 12 weeks) in Ahles et al. (91) compared to Lamport et al. [82], respectively. One study that administered 320 mg/d of purified anthocyanin for 24 weeks, used an entirely different battery of tests (CogTrack) which examined the following cognitive domains (Modified Quality of Episodic Memory (episodic memory), Attentional Intensity Index (Attention), Sustained Attention Index (Attention), Cognitive Reaction Time (Cognitive speed), Attentional Fluctuation Index (Attention), Speed of Memory Retrieval, Quality of Working Memory (working memory)) and did not report significant results for any of the tests.

#### Meta-analysis Results

**Fig. 3:**
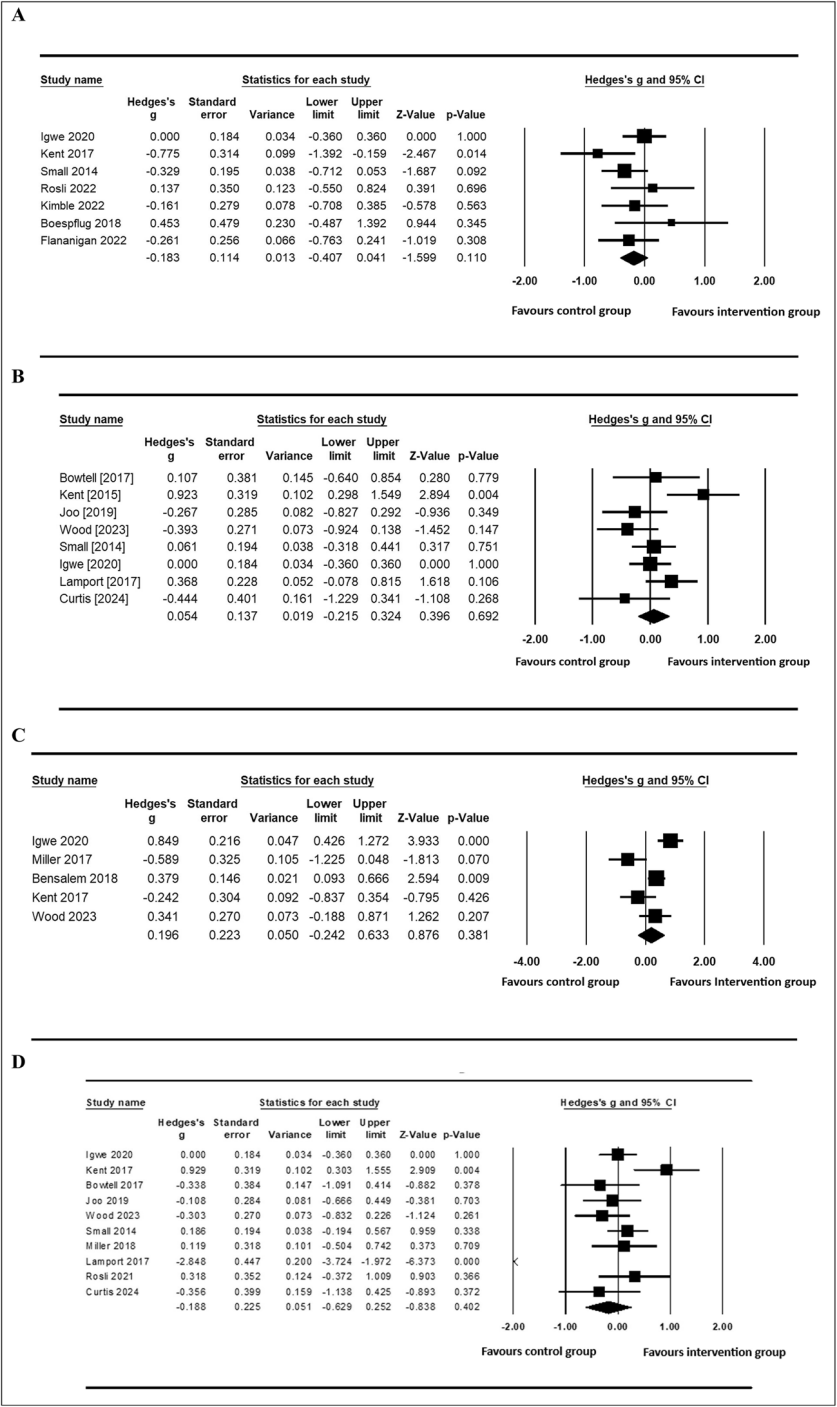
Forest plots from the meta-analysis of clinical trials investigating the effect of anthocyanin on domains of cognition. **A**) Working memory, **B**) Verbal learning memory, **C**) Immediate Memory, **D**) Delayed Memory

Fourteen of the 30 included studies reported sufficient data (including mean values and their respective standard deviation) to be included in the meta-analysis. The domains of verbal memory and working memory had sufficient data to be included in the meta-analysis, with 3 aspects of verbal memory (learning, immediate and delayed) shown as forest plots (Fig. 3). For working memory, meta-analysis of seven studies [80, 81, 85, 87, 89, 91, 111] using the corrected effect size did not demonstrate a statistically significant difference between the intervention and control groups (Working memory Hedges’s g =—0.183, 95% CI = (−0.407, 0.041), *P* = 0.110). There was no statistically significant heterogeneity observed among the studies (Q-value = 7.796, *P*-value = 0.253, I^2^ = 23.041%). Furthermore, nine studies [79–83, 89, 90, 92, 111] that examined learning in the verbal memory domain had non-significant findings (Hedges’s g = 0.054, 95% CI = (−0.215, 0.324), *p* = 0.69); demonstrating moderate heterogeneity, as evidenced through the Cochrane's Q test (Q-value = 14.972, *P*-value = 0.036, I^2^ = 53.245%). Different aspects of verbal memory are commonly assessed within a single one task, allowing for analysis of effects on learning, immediate memory, and delayed memory. Separate meta-analyses encompassing five studies pertaining to immediate memory [30, 80, 86, 88, 90] and 10 studies addressing delayed memory [80–83, 88–90, 92, 109, 111] failed to reach statistical significance as follows: (Hedges’s g = 0.196, 95% CI = (−0.242, 0.633), *p* = 0.38) and (Hedges’s g = −0.188, 95% CI = (−0.629, −0.252), *p* = 0.402, respectively). Significant and substantial heterogeneity was observed in both immediate memory (Q-value = 17.425, *P*-value = 0.002, I^2^ = 77.04) and delayed memory (Q-value = 53.602, *P*-value < 0.005, I^2^ = 83.209), as indicated by the reported statistical values.

### Sensitivity analysis and publication bias

**Fig. 4:**
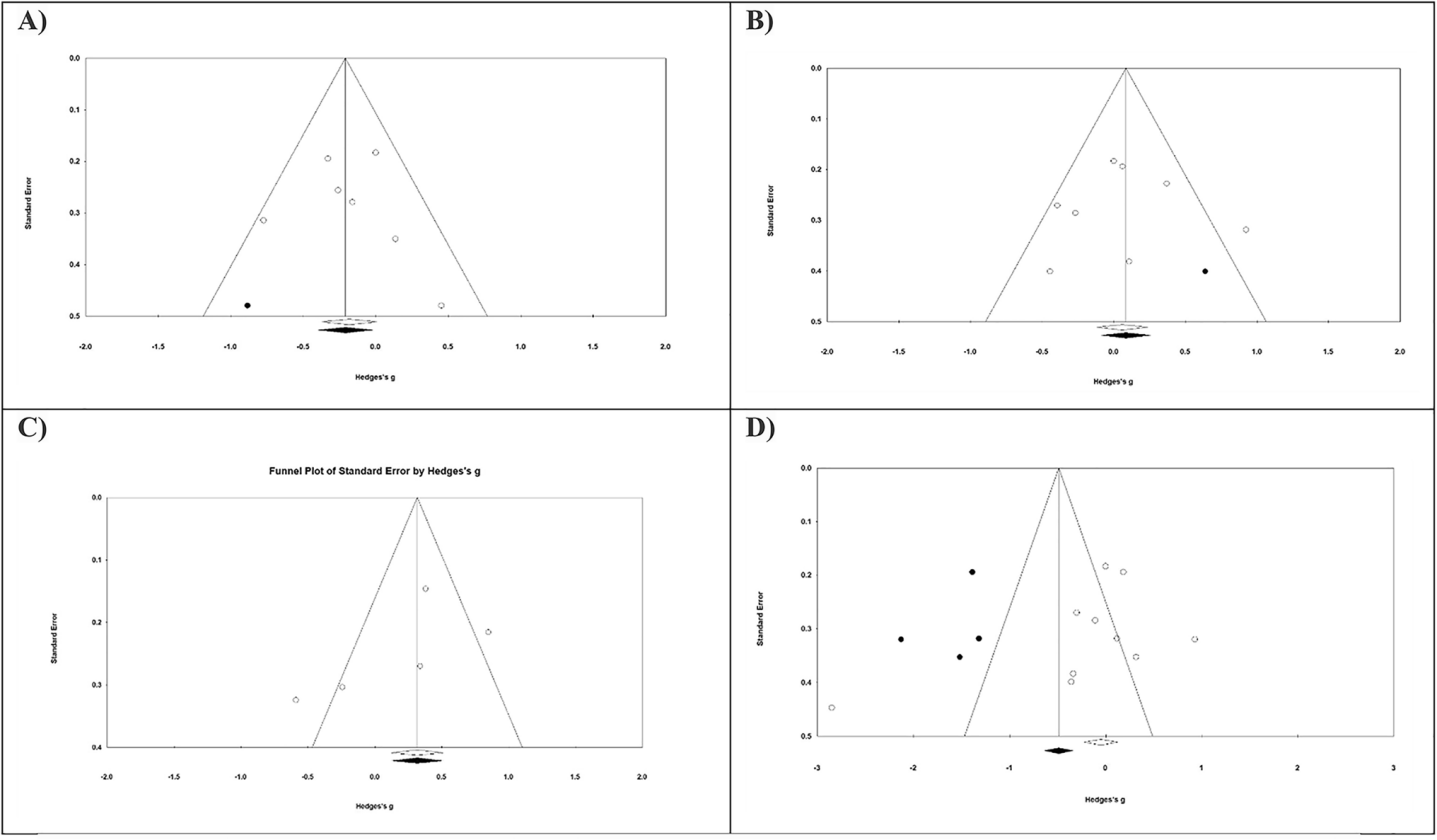
Begg’s funnel plots (with pseudo 95% CIs) depicting effect sizes (Hedges’s g) versus standard errors (SEs) for studies assessing effects of anthocyanin on **A**) Working memory, **B**) Verbal learning memory, **C**) Immediate Memory, **D**) Delayed Memory

The meta-analysis findings were robust to variation in the correlation coefficient of 0.2 and 0.8, underscoring the stability of analyses across diverse outcomes. Additionally, analyses, involving exclusion of individual studies from the meta-analyses did not yield statistically significant effects in the overall results, suggesting no sensitivity. An absence of publication bias was determined through the application of Begg’s and Egger’s asymmetry tests, as illustrated in Fig. 4.

## Discussion

This systematic literature review and meta-analysis evaluated the effect of chronic anthocyanin intake on various cognitive domains across 30 clinical trials in adult populations (18 + y). Overall, evidence from the narrative synthesis shows that anthocyanin consumption improved multiple aspects of cognition and mood, including verbal learning and memory, executive function, working memory, visual spatial function, psychomotor speed, attention, semantic memory, as well as improved symptoms of depression, fatigue and anxiety. This is consistent with previous reviews [27, 30, 114]. Our review, however, is the first to include all adults regardless of their cognitive health status and also consider all sources of anthocyanin. Previous reviews have either focused on healthy middle-aged and older adults [38] or selected a specific single source of anthocyanin [29, 36]. Despite this, there were frequent discrepancies in findings across studies, with no single domain or tool showing a consistent improvement after anthocyanin supplementation. According to our meta-analysis of 14 studies that provided sufficient data on working, verbal learning, immediate and delayed memory, supplementation with anthocyanin in adults did not result in significant changes for any of the cognitive domains. This may be influenced, in part, by the heterogeneity of the included studies.

Although only a small proportion of included studies in this systematic review report improvements in mood measurements [85, 100, 102, 105, 108], a meta-analysis of 10 observational studies suggested an inverse association of fruit rich in anthocyanin intake with risk of depression [115]. Nevertheless, considering that the interventions utilized whole fruits rather than isolated anthocyanin components, the observed effects may be attributed to other unreported flavanols present in the fruit [116]. In addition to anthocyanins, for example blueberries are abundant in flavanols such as quercetin, myricetin, and kaempferol, all of which are linked to cognitive performance and mood. [117]. Furthermore, the benefits of anthocyanin on memory and other cognitive domains, such as executive function [88, 94], psychomotor speed [96], lexical access [97, 99] and attention [84, 85, 89, 100, 106], were also reported in individual studies. These findings are in line with previous studies on the beneficial effects of blueberry supplementation on cognitive performance [27, 114] which further supports the potential effect of anthocyanins in enhancing attention and executive function through their antioxidant properties[118].

The meta-analyses, which did not identify significant effects on specific cognitive domains such as working memory, learning, immediate, and delayed memory, indicated notable heterogeneity. The analysis revealed that there was a lack of substantial or statistically significant heterogeneity concerning working memory even though the meta-analysis was not significant. This observation may be attributed to the consistent utilization of analogous cognitive measurement tools across the included studies or the predominant inclusion of participants with a healthy cognitive status, as delineated in Table 4.

**Table 4:** Meta-analysis showing the overall effect of anthocyanin consumption on working memory, learning memory, immediate and delayed memory

		Meta-analysis		Heterogeneity						
Cognitive tests	No. of effect sizes	Hedges's g (95%CI)	*P* effect	*Q* statistic			*P* within group			*I*^*2*^ (%)
Working memory	7	−0.18 (−0.41, 0.04)	0.11	7.79			0.25			23.04
Study name	Participant characteristics/age	Anthocyanin dose (mg/d)	Cognitive tool				Between group Mean difference (SE)			P-value
Flanagan et al. 2022	Healthy older adults (50–80 years)	340	Digit span backwards				−0.62 (0.60)			0.16
Kimble et al. 2022	Healthy (mean ± SD: 48 ± 6 yrs old)	22.9	N back accuracy				−0.014 (0.02)			0.6
Rosli et al. 2022	Signs of poor cognitive function (45–59 yrs old)	194.1	Digit span backwards				0.4 (1.02)			0.74
Igwe et al. 2020	Healthy (> 55 yrs old)	7.4–10.6	Digit span backwards				0 (0.19)			0.23
Boespflug et al. 2018	MCI (68 –92 yrs old)	269	N back accuracy				0.06 (0.06)			0.08
Kent et al. 2017	Mild-to-moderate dementia Alzheimer’s (> 70 yrs old)	69	Digit span backwards				−0.8 (0.31)			Not significant
Small et al. 2014	Healthy (65–85 yrs old)	225	Digit span backwards				−0.81 (0.48)			> 0.10
Verbal learning memory	**9**	**0.054(−0.215, 0.324)**	**0.69**	**14.97**			**0.03**		**53.24**	
Study name	**Participant characteristics/age**	**Anthocyanin dose (mg/d)**	**Cognitive tool**				**Between group Mean difference (SE)**			***P*-value**
Curtis et al. 2024	mild cognitive impairment (MCI) (mean age 76.33 ± 6.95)	15.9	HVLT free recall				−1.65 (1.47)			0.37
Wood et al. 2023	Healthy (65–80 yrs old)	302 mg/d	Total acquisition				−1.02 (2.29)			0.851
Rosli et al. 2022	Signs of poor cognitive function (45–59 yrs old)	194.1	RAVLT total recall				21.5 (3.91)			0.01
Igwe et al. 2020	Healthy (> 55 yrs old)	7.4–10.6	RVLT total recall				0 (0.19)			0.69
Joo et al. 2019	Subjective memory impairment (> 50 yrs old)	269	Word list memory				−0.54 (0.10)			0.57
Kent et al. 2017	Mild-to-moderate dementia Alzheimer’s (> 70 yrs old)	69	RVLT total recall				5.4 (3.51)			0.01
Bowtell et al. 2017	Healthy (age 67.5 ± 3.0 y)	387	Shopping list accuracy				0.7 (1.20)			Not significant
Lamport et al. 2017	Healthy (aged 40–50 yrs old)	167	VVLT immediate recall				0.6 (0.18)			Not significant
Small et al. 2014	Healthy (65–85 yrs old)	225	immediate recall				0.1 (0.40)			> 0.10
Immediate memory	**5**	**0.20 (−0.24, 0.63)**	**0.38**			**17.43**	**0.002**	**77.04**		
Study name	**Participant characteristics/age**	**Anthocyanin dose (mg/d)**	**Cognitive tool**				**Between group Mean difference (SE)**			**P-value**
Wood et al. 2023	Healthy (65–80 yrs old)	302 mg/d	AVLT Immediate recall				0.59 (2.46)			0.04
Igwe et al. 2020	Healthy (> 55 yrs old)	7.4–10.6	RVLT immediate recall				0.27 (0.06)			Not significant
Bensalem et al. 2018	Healthy (60–70 yrs old)	33.54	VRMFR total correct				−0.54 (0.10)			0.014
Kent et al. 2017	Mild-to-moderate dementia Alzheimer’s (> 70 yrs old)	69	RVLT immediate recall				−0.49 (0.61)			Not reported
Miller et al. 2017	Healthy (age 67.5 ± 3.0 y)	19.2	Shopping list accuracy				−1.3 (0.70)			Not significant
Delayed memory	**10**	**−0.19 (−0.63, 0.25)**	**0.40**		**53.60**		** < 0.005**		**83.21**	
Study name	**Participant characteristics/age**	**Anthocyanin dose (mg/d)**	**Cognitive tool**				**Between group Mean difference (SE)**			**P-value**
Curtis et al. 2024	mild cognitive impairment (MCI) (mean age 76.33 ± 6.95)	15.9	HVLT delayed				−0.95 (1.05)			0.52
Wood et al. 2023	Healthy (65–80 yrs old)	302 mg/d	AVLT Delayed recall 20 m				−0.930 (0.82)			0.029
Rosli et al. 2022	Signs of poor cognitive function (45–59 yrs old)	194.1	RAVLT delayed recall				1.1 (1.21)			0.37
Igwe et al. 2020	Healthy (> 55 yrs old)	7.4–10.6	RAVLT trial 20 m delayed recall				0 (0.0)			Not significant
Joo et al. 2019	Subjective memory impairment (> 50 yrs old)	269	Word list recall				−0.16 (0.42)			0.72
Kent et al. 2017	Mild-to-moderate dementia Alzheimer’s (> 70 yrs old)	69	RAVLT trial 20 m delayed recall				1.72 (0.56)			≤ 0.001
Miller et al. 2017	Healthy (age 67.5 ± 3.0 y)	19.2	CVLT long delay				0.3 (0.80)			Not significant
Bowtell et al. 2017	Healthy (age 67.5 ± 3.0 y)	387	shopping list recall accuracy				−0.8 (0.90)			0.004
Lamport et al. 2017	Healthy (aged 40–50 yrs old)	167	VVLT delayed recall				−0.5 (0.03)			Not significant
Small et al. 2014	Healthy (65–85 yrs old)	225	AVLT Delayed recall				0.63 (0.66)			Not significant

The majority of the studies that reported positive effects in the current review used blueberry (freeze-dried powder (93, 95, 98, 99), concentrate (84) and juice (91)) as the source of anthocyanin. This is in line with the well-documented antioxidant and anti-inflammatory properties of blueberries [57, 87]. Other studies used a combination of fruits (such as mixed berries [107]) which may result in a synergistic effect of different subclasses of flavonoids.

Studies reporting significant improvements in cognitive domains predominantly utilized fruit juice as a carrier for anthocyanins [81, 82, 85, 93, 111]. Notably, only two studies using juice as an intervention did not report significant findings, likely due to their small sample sizes [51, 92]. This observation aligns with a prior systematic review that focused exclusively on juice interventions. This review demonstrated memory improvements in mildly cognitively impaired adults over a 12–15 week period following intervention with various anthocyanin-rich fruit juices [119]. The high levels of flavonoid metabolites, such as anthocyanidins, from flavonoid-rich fruits can cross the blood–brain barrier and influence regions such as the hippocampus, thereby impacting memory and learning. [120].

As previously mentioned, evidence suggests that anthocyanin bioactivity may be enhanced if consumed in a food matrix rather than as a purified supplement, and if consumed in combination with other polyphenols in foods [121]. Therefore, more emphasis could be placed in future studies on understanding the effect of whole foods or diets rather than isolated and encapsulated anthocyanins [98] when investigating targeted outcomes [122]. This is because investigation of single food sources of anthocyanins somewhat limits the translation of the findings to daily nutritional guidance that includes whole-of-diets that contain a range of other anthocyanin rich fruits and vegetables, such as red onions and cabbage. Importantly, anthocyanin content can be influenced by agricultural factors, such as growing conditions of the fruit based on the harvesting season, temperature [123] and soil pH level. Furthermore, different procedures for food processing can affect anthocyanin content of the carriers. For example, heating anthocyanin-rich plums for the purpose of jam-making caused a 70% reduction in total anthocyanin content [124]. Storage conditions can also affect anthocyanin degradation [125]. Surprisingly, most studies do not report the anthocyanin content provided as the intervention over time, which may be problematic when considering longer treatment periods are generally required to induce an effect. The dose administered across the studies included in the present review varied greatly, between 10 and 598 mg/day. However, no dose response effects were observed due to a lack of studies that compared the impacts of differing doses of anthocyanins provided from the same food sources to individuals with similar characteristics over time. This finding is further corroborated by recent meta-analyses [37, 38] which did not identify a consistent pattern regarding the type and dosage of anthocyanin supplementation across these studies.

Individual variability in anthocyanin bioavailability may have contributed to the varied effects observed in the cited studies, therefore, pharmacokinetic studies (including different populations across age and cognitive function categories) are required. Most significant cognitive improvements that were observed in the studies included in the present review were from those conducted in healthy young participants. It has been well established that human digestive and absorptive efficiencies decline with advancing age [30] and that prescribed medication can affect gut microbiota [126]. It is feasible that anthocyanin metabolite bioavailability may be decreased in older adults, both peripherally and in the brain. Furthermore, individuals with impaired cognitive function resulting from vascular and nonvascular dementia and Alzheimer’s disease may respond differently to anthocyanin administration compared to healthy adults. Another possible explanation is that oxidative stress levels are generally lower in young, healthy adults; thus, additional antioxidant support from anthocyanins may enhance cognitive function, resulting in higher performance and scores on cognitive assessments. In contrast, older adults with cognitive impairments often have extensive oxidative damage accumulated over time [127, 128], which may exceed the capacity of anthocyanins to counteract oxidative damage effectively.

This review included studies that administered interventions for at least a week or more and the shortest study included had a duration of 5 weeks. However, improvements were noted up to 24 weeks of intervention. Overall, studies with a duration of 12 weeks or longer showed significant benefits of anthocyanin consumption on cognition, suggesting that anthocyanin supplementation may exhibit strong neurocognitive properties if consumed over the longer term [129, 130]. This finding is similar to previous literature which suggests that polyphenol administration needs to have a longer duration (12–24 months) before it shows significant health benefits on cognition [131]. The mechanisms by which anthocyanins can improve cognitive function are beginning to be better understood. Studies report that anthocyanins can potentially influence monoaminergic signalling in the brain, as anthocyanins have an affinity for signalling components such as, monoamine oxidase A and B (MAO-A and MAO-B) enzymes [132] and the dopaminergic D2 receptor [133]. Furthermore, anthocyanins are protective of dopaminergic neurons in pre-clinical models of Parkinson’s disease [134], which could relate to their effects on executive functions and attention; however, further investigation is required.

### Strength and Limitations

The strengths of this study are multifaceted. Firstly, the review encompassed studies with diverse populations, ranging from healthy individuals to those with populations experiencing mild to moderate memory decline, Alzheimer’s disease, self-reported depression, metabolic syndrome (including overweight/obesity and insulin resistance) and diagnosed mild cognitive impairment. The present review also included studies of adults across various age groups, including younger adults, middle-aged and older adults. The broad inclusion of the adult population enhances the generalizability of the findings. Secondly, the paper evaluated the impact of anthocyanins on a wide array of cognitive domains and mood measures, with extensive coverage providing a more comprehensive understanding of the potential neuropsychological benefits of anthocyanins than previously published. Moreover, this study not only highlighted the potential cognitive and mood benefits of anthocyanins but also critically examined the substantial heterogeneity in the results. By identifying uncontrollable sources of heterogeneity, such as inconsistencies in effect sizes, anthocyanin dose and dietary source, treatment duration and participant health status, and variance in cognition and mood measures across the studies, the present review underscores the necessity for more standardized future research. Additionally, this review discusses the practical implications of anthocyanin consumption for cognitive health and the challenges in translating current research findings into dietary recommendations due to the heterogeneity of findings. This focus on real-world applications enhances the current research relevance and utility.

Limitations of the current review include inconsistencies regarding test instrumentation and experimental methodology between studies which limited the opportunity to reach a definitive conclusion regarding the effect of anthocyanins on these domains of cognition in the current meta-analysis. It is noteworthy that performance in cognitive tasks does not rely solely on measuring a single domain. There are a lack of studies using the same tests to assess a particular domain, which limited the scope of this meta-analysis. This limitation has been recognized previously and addressed in a methodological review by de Jager et al. [135]. The varying difficulty levels of cognitive assessments may have also presented challenges for the meta-analysis. Evidence suggests that more basic cognitive abilities, such as attention and processing speed, are generally less complex than higher-order functions like learning and memory [136]. This review did not account for task difficulty across cognitive domains, including memory, attention, executive function, and psychomotor performance, partly because many studies did not justify their choice of specific cognitive tests within the same test domain. Although control groups in the included studies generally received a well-matched placebo that was devoid of anthocyanins and had minimal polyphenolic content, participants' usual background dietary intake of these compounds was not accounted for. The lack of strict control over participants' habitual intake of anthocyanins and other polyphenols, in addition to inadequate monitoring of adherence to the prescribed treatments are important limitations to consider.

It is important to acknowledge that more studies are required to identify populations of individuals that are most likely to benefit from anthocyanin supplementation and to identify the doses of anthocyanins and food sources that are most effective at inducing cognitive and mood benefits. There is a need for more well-designed clinical trials that apply consistent, standardized measurements of cognitive and mood domains by ensuring clear rationales for selection of specific cognitive tests, together with an assessment of background dietary intake of participants and reporting of anthocyanin concentration in intervention foods over the duration of the study period.

## Conclusion

This systematic literature review of randomised clinical trials that assessed the impact of anthocyanins, provided either as food or in supplemental form, on a range of cognitive domains, including mood, in adult populations (> 18y) suggests several beneficial effects. However, the heterogeneity found in the meta-analyses mitigated these effects for working memory, verbal learning memory, immediate memory and delayed memory. Despite efforts made to reduce heterogeneity by restricting the meta-analyses to studies employing a congruent cognitive task as the domain measure, uncontrollable sources of heterogeneity persisted. Notably, there seems to be little explanation for the observed data variations. For instance, studies demonstrating more substantial effects did not uniformly use larger dosages, nor find more pronounced effects in cognitively impaired participants. Therefore, further well-designed studies using a standardized method of cognitive domain measurement are required to inform dietary guidance for both healthy and cognitively impaired populations.

## Supplementary Information

Below is the link to the electronic supplementary material.

Supplementary file1 (DOCX 18 KB)

## Data Availability

Data is provided within the manuscript or supplementary information files.
