# Supplementary material for: The Effect of Anthocyanins on Cognition: A Systematic Review and Meta-analysis of Randomized Clinical Trial Studies in Cognitively Impaired and Healthy Adults
Source: Curr Nutr Rep. 2025 Jan 29;14(1):23. doi: 10.1007/s13668-024-00595-z (PMC11775034; doi:10.1007/s13668-024-00595-z)
Supplement: Supplementary file 1 — Supplementary file1 (DOCX 18 KB) [file 13668_2024_595_MOESM1_ESM.docx]

**Supplementary material: Key words used in the search.**

Three groups of medical subject headings (MeSH) and non-MeSH keywords were selected to search the databases, as follows.

| PUBMED |
| --- |
| (anthocyanin*[MeSH] OR anthocyanin*[tiab] OR Leucoanthocyanidins[tiab] OR Anthocyanidin*[tiab] OR Anthocyanidin[tiab] OR Cyanidin*[tiab] OR Delphinidin*[tiab] OR Malvidin*[tiab] OR Pelargonidin*[tiab] OR Peonidin*[tiab] OR Petunidin*[tiab]) AND (intervention*[tiab] OR pilot*[tiab] OR trial[tiab] OR randomized[tiab] OR random*[tiab] OR randomly[tiab] OR non-random*[tiab] OR nonrandom*[tiab] OR placebo[tiab] OR assignment*[tiab] OR "clinical trial*"[publication type] OR RCT[tiab] OR "Clinical Trials as Topic"[MeSH] OR cross-over[tiab] OR "cross over"[tiab] OR parallel[tiab]) AND (Cognition[MeSH] OR cognitions[tiab] OR "cognitive function"[tiab] OR "cognitive functions"[tiab] OR memory[MeSH] OR memory[tiab] OR "episodic memory"[tiab] OR "long-term memory"[tiab] OR "Memory Consolidation"[tiab] OR learning[tiab] OR Phenomenography[tiab] OR "mental process*"[tiab] OR "mental health"[MeSH] OR "mental health"[tiab] OR Depression*[MeSH] OR Depression*[tiab] OR depress*[tiab] OR antidepress*[tiab] OR cognit*[tiab] OR "Depressive Symptom*"[tiab] OR "Depressive Symptom*"[tiab] OR "Depressive symptom*"[tiab] OR "Emotional Depression*"[tiab] OR awareness[tiab] OR "cognitive dissonance"[tiab] OR comprehension[tiab] OR "cognitive reserve"[tiab] OR consciousness[tiab] OR mood*[tiab] OR anxiety[MeSH] OR anxiety[tiab] OR Stress[tiab] OR "stress disorder*"[MeSH] OR "stress disorder*"[tiab] OR "Brain-derived neurotrophic factor"[tiab] OR BDNF[tiab] OR "neuroprotective effect*"[tiab] OR "neuroprotective measure*"[tiab] OR "Trail Making test"[tiab] OR TMT[tiab] OR "Alzheimer’s Disease Assessment Scale-cognitive"[tiab] OR "ADAS-Cog"[tiab] OR "Mini-Mental State Examination"[tiab] OR MMSE[tiab] OR "Wechsler Adult Intelligence Scale"[tiab] OR WAIS[tiab] OR "Center for Epidemiologic Studies - Depression Scale"[tiab] OR CES-D[tiab] OR "The Beck Depression Inventory"[tiab] OR BDI[tiab] OR "Edinburgh Postnatal Depression Scale"[tiab] OR EPDS[tiab] OR "State Trait Anxiety Inventory"[tiab] OR STAI[tiab] OR "Hamilton Anxiety Rating Scale"[tiab] OR HAM-A[tiab] OR "Beck Anxiety Inventory"[tiab] OR BAI[tiab] OR "Profile of Mood States"[tiab] OR POMS[tiab] OR "visual analogue scale"[tiab] OR VAS[tiab] ) NOT (mouse[tiab] OR mice[tiab] OR rats[tiab] OR in-vitro[tiab] OR "in vitro"[tiab] OR Pig[tiab] OR Rabbit[tiab] OR Rooster[tiab] OR cell[tiab] OR cells[tiab] OR cow[tiab] OR cows[tiab]) |

| SCOPUS |
| --- |
| (TITLE-ABS-KEY(Anthocyanin*) OR TITLE-ABS-KEY(Leucoanthocyanidin*) OR TITLE-ABS-KEY(Anthocyanidin*) OR TITLE-ABS-KEY(Cyanidin*) OR TITLE-ABS-KEY(Delphinidin*) OR TITLE-ABS-KEY(Malvidin*) OR TITLE-ABS-KEY(Pelargonidin*) OR TITLE-ABS-KEY(Peonidin*) OR TITLE-ABS-KEY(Petunidin*)) AND (TITLE-ABS-KEY(intervention*) OR TITLE-ABS-KEY(pilot*) OR TITLE-ABS-KEY(trial) OR TITLE-ABS-KEY(randomized) OR TITLE-ABS-KEY(random*) OR TITLE-ABS-KEY(non-random*) OR TITLE-ABS-KEY(nonrandom*) OR TITLE-ABS-KEY(placebo) OR TITLE-ABS-KEY(assignment*) OR TITLE-ABS-KEY(“clinical trial*”) OR TITLE-ABS-KEY(RCT) OR TITLE-ABS-KEY(“Clinical Trials as Topic”) OR TITLE-ABS-KEY(cross-over) OR TITLE-ABS-KEY(parallel)) AND (TITLE-ABS-KEY(Cognition*) OR TITLE-ABS-KEY(“cognitive function*”) OR TITLE-ABS-KEY(memory) OR TITLE-ABS-KEY(“episodic memory”) OR TITLE-ABS-KEY(“long-term memory”) OR TITLE-ABS-KEY(“Memory Consolidation”) OR TITLE-ABS-KEY(learning) OR TITLE-ABS-KEY(“mental health”) OR TITLE-ABS-KEY(Phenomenography) OR TITLE-ABS-KEY(“mental process*”) OR TITLE-ABS-KEY(Depression*) OR TITLE-ABS-KEY(depress*) OR TITLE-ABS-KEY(antidepress*) OR TITLE-ABS-KEY(cognit*) OR TITLE-ABS-KEY(“Depressive Symptom*”) OR TITLE-ABS-KEY(“Emotional Depression*”) OR TITLE-ABS-KEY(awareness) OR TITLE-ABS-KEY(“cognitive dissonance”) OR TITLE-ABS-KEY(comprehension) OR TITLE-ABS-KEY(“cognitive reserve”) OR TITLE-ABS-KEY(consciousness) OR TITLE-ABS-KEY(mood*) OR TITLE-ABS-KEY(anxiety) OR TITLE-ABS-KEY(Stress) OR TITLE-ABS-KEY(“stress disorder*”) OR TITLE-ABS-KEY(“Brain-derived neurotrophic factor”) OR TITLE-ABS-KEY(BDNF) OR TITLE-ABS-KEY(“neuroprotective effect*”) OR TITLE-ABS-KEY(“neuroprotective measure*”) OR TITLE-ABS-KEY(“Wisconsin Card Classification Test”) OR TITLE-ABS-KEY(WCST) OR TITLE-ABS-KEY(“Reys Auditory Verbal Learning Task”) OR TITLE-ABS-KEY(RAVLT) OR TITLE-ABS-KEY(“Trail Making test”) OR TITLE-ABS-KEY(TMT) OR TITLE-ABS-KEY(“Alzheimer’s Disease Assessment Scale-cognitive”) OR TITLE-ABS-KEY(“ADAS-Cog”) OR TITLE-ABS-KEY(“Mini-Mental State Examination”) OR TITLE-ABS-KEY(MMSE) OR TITLE-ABS-KEY(“Wechsler Adult Intelligence Scale”) OR TITLE-ABS-KEY(WAIS) OR TITLE-ABS-KEY(“Center for Epidemiologic Studies - Depression Scale”) OR TITLE-ABS-KEY(CES-D) OR TITLE-ABS-KEY(“The Beck Depression Inventory”) OR TITLE-ABS-KEY(BDI) OR TITLE-ABS-KEY(“Edinburgh Postnatal Depression Scale”) OR TITLE-ABS-KEY(EPDS) OR TITLE-ABS-KEY(“State Trait Anxiety Inventory”) OR TITLE-ABS-KEY(STAI) OR TITLE-ABS-KEY(“Hamilton Anxiety Rating Scale”) OR TITLE-ABS-KEY(HAM-A) OR TITLE-ABS-KEY(“Beck Anxiety Inventory”) OR TITLE-ABS-KEY(BAI) OR TITLE-ABS-KEY(“Profile of Mood States”) OR TITLE-ABS-KEY(POMS) OR TITLE-ABS-KEY(“Positive and Negative Activation Schedule”) OR TITLE-ABS-KEY(PANAS) OR TITLE-ABS-KEY(“visual analogue scale”) OR TITLE-ABS-KEY(VAS) ) AND NOT (TITLE-ABS-KEY(mouse) OR TITLE-ABS-KEY(mice) OR TITLE-ABS-KEY(rats) OR TITLE-ABS-KEY(in-vitro) OR TITLE-ABS-KEY(“in vitro”) OR TITLE-ABS-KEY(Pig) OR TITLE-ABS-KEY(Rabbit) OR TITLE-ABS-KEY(Rooster) OR TITLE-ABS-KEY(cell) OR TITLE-ABS-KEY(cells) OR TITLE-ABS-KEY(cow) OR TITLE-ABS-KEY(cows)) |

| WEB OF SCIENCE (ISI) |
| --- |
| #1=  (TS= (Anthocyanin*) OR TS=(Leucoanthocyanidin*) OR TS=(Anthocyanidin*) OR TS=(Cyanidin*) OR TS=(Delphinidin*) OR TS=(Malvidin*) OR TS=(Pelargonidin*) OR TS=(Peonidin*) OR TS=(Petunidin*)) AND (TS=(intervention*) OR TS=(pilot*) OR TS=(trial) OR TS=(randomized) OR TS=(random*) OR TS=(non-random*) OR TS=(placebo) OR TS=(assignment*) OR TS=(“clinical trial*”) OR TS=(RCT) OR TS=(“Clinical Trials as Topic”) OR TS=(“cross over”) OR TS=(cross-over) OR TS=(parallel)) AND (TS=(Cognition*) OR TS=(“cognitive function*”) OR TS=(memory) OR TS=(“episodic memory”) OR TS=(“long-term memory”) OR TS=(“Memory Consolidation”) OR TS=(learning) OR TS=(“mental health”) OR TS=(Phenomenography) OR TS=(“mental process*”) OR TS=(Depression*) OR TS=(depress*) OR TS=(antidepress*) OR TS=(cognit*) OR TS=(“Depressive Symptom*”) OR TS=(“Emotional Depression*”) OR TS=(awareness) OR TS=(“cognitive dissonance”) OR TS=(comprehension) OR TS=(“cognitive reserve”) OR TS=(consciousness) OR TS=(mood*) OR TS=(anxiety) OR TS=(Stress) OR TS=(“stress disorder*”) OR TS=(“Brain-derived neurotrophic factor”) OR TS=(BDNF) OR TS=(“neuroprotective effect*”) OR TS=(“neuroprotective measure*”) OR TS=(“Wisconsin Card Classification Test”) OR TS=(WCST) OR TS=(“Reys Auditory Verbal Learning Task”) OR TS=(RAVLT) OR TS=(“Trail Making test”) OR TS=(TMT) OR TS=(“Alzheimer’s Disease Assessment Scale-cognitive subscale”) OR TS=(“ADAS-Cog”) OR TS=(“Mini-Mental State Examination”) OR TS=(MMSE) OR TS=(“Wechsler Adult Intelligence Scale”) OR TS=(WAIS) OR TS=(“Center for Epidemiologic Studies - Depression Scale”) OR TS=(CES-D) OR TS=(“The Beck Depression Inventory”) OR TS=(BDI) OR TS=(“Edinburgh Postnatal Depression Scale”) OR TS=(EPDS) OR TS=(“State Trait Anxiety Inventory”) OR TS=(STAI) OR TS=(“Hamilton Anxiety Rating Scale”) OR TS=(HAM-A) OR TS=(“Beck Anxiety Inventory”) OR TS=(BAI) OR TS=(“Profile of Mood States”) OR TS=(POMS) OR TS=(“Positive and Negative Activation Schedule”) OR TS=(PANAS) OR TS=(“visual analogue scale”) OR TS=(VAS)) NOT (TS=(mouse) OR TS=(mice) OR TS=(rats) OR TS=(in-vitro) OR TS=(“in vitro”) OR TS=(Pig) OR TS=(Rabbit) OR TS=(Rooster) OR TS=(cell*) OR TS=(cow*))  #2=  (TI=(Anthocyanin*) OR TI=(Leucoanthocyanidin*) OR TI=(Anthocyanidin*) OR TI=(Cyanidin*) OR TI=(Delphinidin*) OR TI=(Malvidin*) OR TI=(Pelargonidin*) OR TI=(Peonidin*) OR TI=(Petunidin*)) AND (TI=(intervention*) OR TI=(pilot*) OR TI=(trial) OR TI=(randomized) OR TI=(random*) OR TI=(non-random*) OR TI=(placebo) OR TI=(assignment*) OR TI=(“clinical trial*”) OR TI=(RCT) OR TI=(“Clinical Trials as Topic”) OR TI=(“cross over”) OR TI=(cross-over) OR TI=(parallel)) AND (TI=(Cognition*) OR TI=(“cognitive function*”) OR TI=(memory) OR TI=(“episodic memory”) OR TI=(“long-term memory”) OR TI=(“Memory Consolidation”) OR TI=(learning) OR TI=(“mental health”) OR TI=(Phenomenography) OR TI=(“mental process*”) OR TI=(Depression*) OR TI=(depress*) OR TI=(antidepress*) OR TI=(cognit*) OR TI=(“Depressive Symptom*”) OR TI=(“Emotional Depression*”) OR TI=(awareness) OR TI=(“cognitive dissonance”) OR TI=(comprehension) OR TI=(“cognitive reserve”) OR TI=(consciousness) OR TI=(mood*) OR TI=(anxiety) OR TI=(Stress) OR TI=(“stress disorder*”) OR TI=(“Brain-derived neurotrophic factor”) OR TI=(BDNF) OR TI=(“neuroprotective effect*”) OR TI=(“neuroprotective measure*”) OR TI=(“Wisconsin Card Classification Test”) OR TI=(WCST) OR TI=(“Reys Auditory Verbal Learning Task”) OR TI=(RAVLT) OR TI=(“Trail Making test”) OR TI=(TMT) OR TI=(“Alzheimer’s Disease Assessment Scale-cognitive subscale”) OR TI=(“ADAS-Cog”) OR TI=(“Mini-Mental State Examination”) OR TI=(MMSE) OR TI=(“Wechsler Adult Intelligence Scale”) OR TI=(WAIS) OR TI=(“Center for Epidemiologic Studies - Depression Scale”) OR TI=(CES-D) OR TI=(“The Beck Depression Inventory”) OR TI=(BDI) OR TI=(“Edinburgh Postnatal Depression Scale”) OR TI=(EPDS) OR TI=(“State Trait Anxiety Inventory”) OR TI=(STAI) OR TI=(“Hamilton Anxiety Rating Scale”) OR TI=(HAM-A) OR TI=(“Beck Anxiety Inventory”) OR TI=(BAI) OR TI=(“Profile of Mood States”) OR TI=(POMS) OR TI=(“Positive and Negative Activation Schedule”) OR TI=(PANAS) OR TI=(“visual analogue scale”) OR TI=(VAS)) NOT (TI=(mouse) OR TI=(mice) OR TI=(rat) OR TI=(in-vitro) OR TI=(“in vitro”) OR TI=(Pig) OR TI=(Rabbit) OR TI=(Rooster) OR TI=(cell*) OR TI=(cow*))  #1 OR #2 |
